# HDAC4 Reduction: A Novel Therapeutic Strategy to Target Cytoplasmic Huntingtin and Ameliorate Neurodegeneration

**DOI:** 10.1371/journal.pbio.1001717

**Published:** 2013-11-26

**Authors:** Michal Mielcarek, Christian Landles, Andreas Weiss, Amyaouch Bradaia, Tamara Seredenina, Linda Inuabasi, Georgina F. Osborne, Kristian Wadel, Chrystelle Touller, Rachel Butler, Janette Robertson, Sophie A. Franklin, Donna L. Smith, Larry Park, Paul A. Marks, Erich E. Wanker, Eric N. Olson, Ruth Luthi-Carter, Herman van der Putten, Vahri Beaumont, Gillian P. Bates

**Affiliations:** 1 Department of Medical and Molecular Genetics, King's College London, London, United Kingdom; 2 Novartis Institutes for BioMedical Research, Neuroscience Discovery, Basel, Switzerland; 3 Neuroservice, Aix en Provence, France; 4 Brain Mind Institute, Ecole Polytechnique Federale de Lausanne, Lausanne, Switzerland; 5 CHDI Management Inc./CHDI Foundation, Los Angeles, California, United States of America; 6 Cell Biology Program, Memorial Sloan-Kettering Cancer Center, New York, New York, United States of America; 7 Neuroproteomics, Max Delbrueck Center for Molecular Medicine, Berlin, Germany; 8 Department of Molecular Biology, Southwestern University, Dallas, Texas, United States of America; Whitehead Institute, United States of America

## Abstract

Histone deacetylase (HDAC) 4 is a transcriptional repressor that contains a glutamine-rich domain. We hypothesised that it may be involved in the molecular pathogenesis of Huntington's disease (HD), a protein-folding neurodegenerative disorder caused by an aggregation-prone polyglutamine expansion in the huntingtin protein. We found that HDAC4 associates with huntingtin in a polyglutamine-length-dependent manner and co-localises with cytoplasmic inclusions. We show that HDAC4 reduction delayed cytoplasmic aggregate formation, restored *Bdnf* transcript levels, and rescued neuronal and cortico-striatal synaptic function in HD mouse models. This was accompanied by an improvement in motor coordination, neurological phenotypes, and increased lifespan. Surprisingly, HDAC4 reduction had no effect on global transcriptional dysfunction and did not modulate nuclear huntingtin aggregation. Our results define a crucial role for the cytoplasmic aggregation process in the molecular pathology of HD. HDAC4 reduction presents a novel strategy for targeting huntingtin aggregation, which may be amenable to small-molecule therapeutics.

## Abstract 2

HDAC4 histone deacetylase is found to associate with huntingtin in a polyQ-length dependent manner. Reduction of HDAC4 levels in mouse models of Huntington's disease (HD) delays cytoplasmic aggregation in the brain and improves the molecular pathology of HD, providing a potential new therapeutic target.

## Author Summary

Huntington's disease (HD) is a late-onset neurodegenerative disorder caused by protein-folding defects in the huntingtin protein. Mutations in huntingtin can result in extra-long tracts of the amino acid glutamine, resulting in aberrant interactions with other proteins and also causing huntingtin proteins to self-associate and -aggregate. The pathology of HD is therefore associated with nuclear and cytoplasmic aggregates. HDAC4 is a histone deacetylase protein traditionally associated with roles in transcription repression. The HDAC4 protein contains a glutamine-rich domain and in this work we find that HDAC4 associates with huntingtin in a polyglutamine-length-dependent manner and that these proteins co-localise in cytoplasmic inclusions. Importantly, reducing HDAC4 levels delays cytoplasmic aggregate formation and rescues neuronal and cortico-striatal synaptic function in mouse models of HD. In addition, we observe improvements in motor coordination and neurological phenotypes, as well as increased lifespan in these mice. Nuclear huntingin aggregates or transcription regulation, however, remained unaffected when HDAC4 levels were reduced to enable these effects. Our results thus provide valuable insight into separating cytoplasmic and nuclear pathologies, and define a crucial role for cytoplasmic aggregations in HD progression. HDAC4 reduction presents a novel strategy for alleviating the toxicity of huntingtin protein aggregation, thereby influencing the molecular pathology of Huntington's disease. As there are currently no disease-modifying therapeutics available for Huntington's disease, we hope that this HDAC4-mediated regulation may be amenable to small-molecule therapeutics.

## Introduction

Huntington's disease (HD) is a progressive, inherited neurological disorder characterized by severe motor, cognitive, behavioural, and physiological dysfunction for which there is no effective disease-modifying treatment [1]. The disease is caused by the expansion of a CAG repeat to more than 35 CAGs within exon 1 of the *HTT* gene. At the molecular level, mutant huntingtin (HTT) containing an expanded polyQ stretch has a propensity to self-aggregate to produce a wide-range of oligomeric species and insoluble aggregates and exerts a gain of toxic function through aberrant protein–protein interactions [2]. Therefore, as with other neurodegenerative diseases such as Alzheimer's disease, Parkinson's disease, and the prion diseases, the polyglutamine (polyQ) disorders including HD are associated with the accumulation of misfolded proteins leading to neuronal dysfunction and cell death.

Transcriptional dysregulation is part of the complex molecular pathogenesis of HD, to which abnormal histone acetylation and chromatin remodelling may contribute [3]. The imbalance in histone acetylation was proposed to be caused by the inactivation of histone acetyltransferases, which led to the pursuit of histone deacetylases (HDACs) as HD therepeutic targets [4],[5]. There are 11 mammalian Zn^2+^-dependent HDACs divided into three groups based on structural and functional similarities: class I (HDACs: 1, 2, 3, 8), class IIa (HDACs: 4, 5, 7, 9), class IIb (HDACs: 6, 10), and HDAC11 as class IV [6]. Initial genetic and pharmacological studies performed in flies, worms, and HD mouse models have suggested that HDAC inhibitors may have a significant therapeutic potential [4],[5].

The preclinical evaluation of the HDAC inhibitor suberoylanilide hydroxamic acid (SAHA) demonstrated a dramatic improvement in the motor impairment that develops in the R6/2 HD mouse model [7]. Initially, SAHA was shown to inhibit class I and II HDACs at nanomolar concentrations, although it is predominantly a class I inhibitor [8]. More recently, SAHA was shown to lead to the degradation of HDACs 4 and 5 via RANBP2-mediated proteasome degradation in cancer cell lines [9]. Following on from this, we demonstrated that in addition to its deacetylase activity and the known effect on decreasing *Hdac7* mRNA levels [10], SAHA treatment results in a reduction in HDAC2 and HDAC4 in brain regions of both WT and R6/2 mice, without affecting their transcript levels *in vivo*. This was associated with a reduction in aggregate load and the restoration of cortical *Bdnf* transcript levels in R6/2 mice [11].

It is well-established that HDAC4 acts as a transcriptional repressor that shuttles between the nucleus and cytoplasm. Phosphorylated HDAC4 is retained in the cytoplasm through its association with 14-3-3 proteins [12]. The N-terminal region of HDAC4 contains a MEF2 binding site and represses the transcription of MEF2-dependent genes important in the regulation of neuronal cell death [13]. In this context, MEF2 can act as a neuronal survival factor, and its inhibition has been linked to the death of neurons in several cell culture systems [14]. Crystallization of the N-terminal domain of HDAC4 suggested that HDAC4 might have the propensity to self-aggregate through its glutamine-rich domains, consistent with cell-culture studies [15]. Interestingly, regions containing high glutamine content in proteins have been observed to facilitate interactions with other glutamine-rich proteins, leading to the spontaneous assembly of insoluble toxic amyloid-like structures in mammalian cells [16].

In this study, we identified a novel mechanism by which HDACs can modify HD pathogenesis *in vivo* and found that HDAC4 reduction delays the HTT aggregation process. We demonstrated that HDAC4 associates with mutant exon 1 and full-length HTT *in vivo* in a polyQ-length-dependent manner and co-localizes with cytoplasmic inclusions in the brains of HD mouse models. HDAC4 knock-down inhibited aggregate formation in both the R6/2 (N-terminal fragment) and *Hdh*Q150 (full-length knock-in) HD mouse models. This delay in aggregation occurred in the cytoplasm, consistent with the subcellular localisation of HDAC4 in mouse brain. We found no evidence for HDAC4 translocation to the nucleus during disease progression, and HDAC4 knock-down had no effect on HTT aggregation in the nucleus and no impact on global transcriptional dysregulation. HDAC4 reduction led to a marked restoration of the membrane properties of medium spiny neurons (MSNs) and of corticostriatal synaptic transmission. This was associated with an improvement in neurological phenotypes and extended survival. These data provide a clear demonstration that cytoplasmic pathogenic mechanisms contribute to HD-related neurodegenerative phenotypes and identify HDAC4 as a therapeutic target for HD.

## Results

### Molecular Characterisation of *Hdac4* Knock-Down in Mouse Models of HD

**Figure 1:**
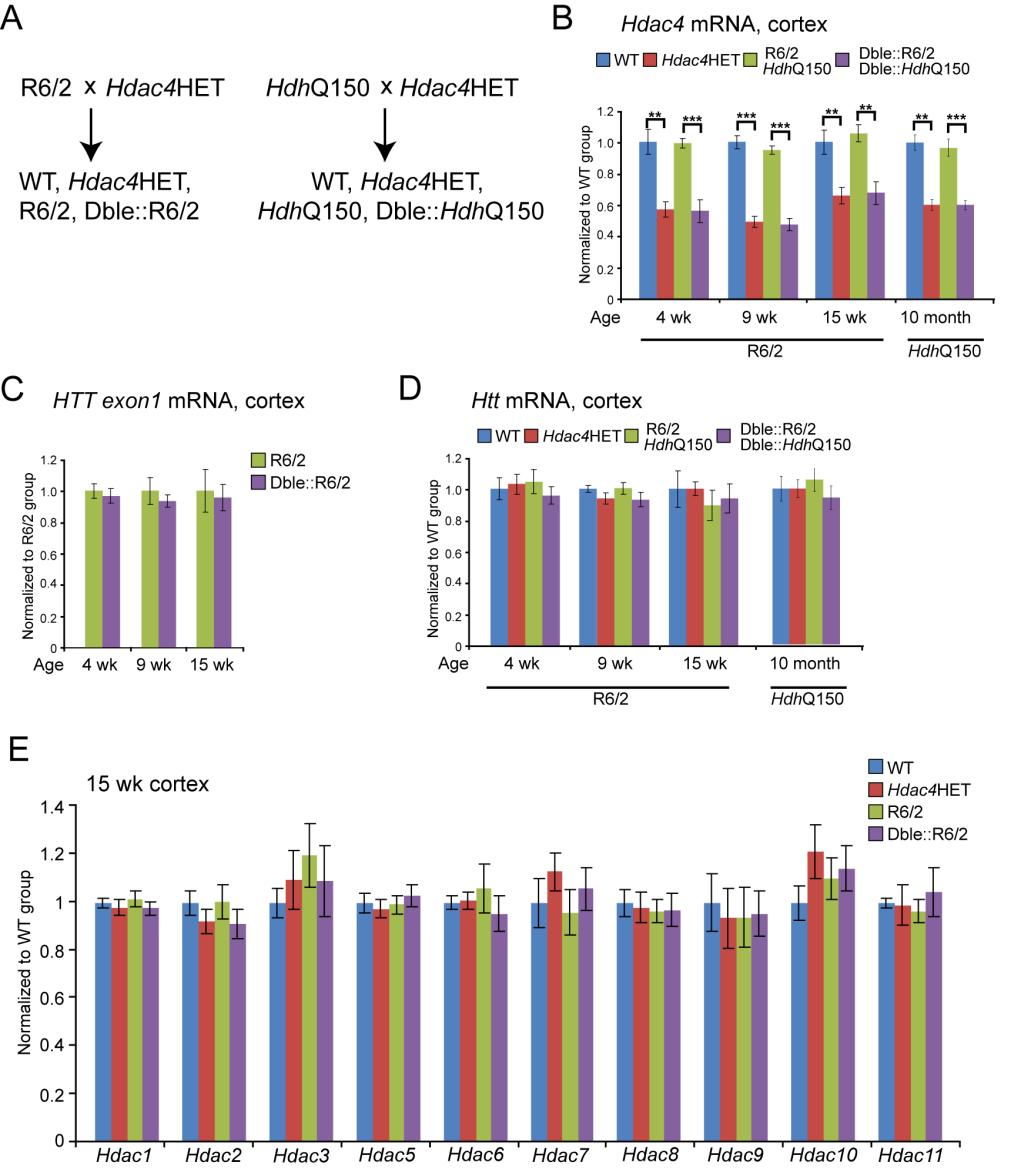
*Hdac4* reduction does not alter the expression levels of *HTT* exon 1, endogenous *Htt*, or other *Hdacs*. (A) Breeding scheme used to reduce *Hdac4* levels in both R6/2 and heterozygous *Hdh*Q150 mice. WT, wild type; *Hdac4*HET, *Hdac4*KO heterozygotes; Dble::R6/2, R6/2 mice heterozygous for *Hdac4*KO; Dble::*Hdh*Q150, *Hdh*Q150 mice heterozygous for *Hdac4*KO. (B) *Hdac*4 transcript levels were decreased in *Hdac*4HET, Dble::R6/2, and Dble::*Hdh*Q150 mice as measured by Taqman qPCR. (C) Taqman qPCR showed that *HTT* exon 1 transgene levels did not differ between R6/2 and Dble::R6/2 mice. (D) Taqman qPCR showed that the expression of endogenous *Htt* was equivalent between WT and *Hdac4*HETs and did not change when *Hdac4* was knocked down in R6/2 or *Hdh*Q150 mice. (E) The transcript levels of other *Hdacs* were equivalent to WT levels in *Hdac*4HET, R6/2, and Dble::R6/2 mice as determined by Taqman qPCR. All Taqman qPCR values were normalized to the geometric mean of three housekeeping genes: *Atp5b*, *Canx*, and *Rpl13a*. Error bars are SEM using Student's *t* test (*n* = 8). **p<0.01; ***p<0.001.

In order to investigate whether HDAC4 is involved in the molecular pathogenesis of HD, we used a genetic approach to reduce HDAC4 levels in both the R6/2 and *Hdh*Q150 knock-in HD mouse models. R6/2 mice are transgenic for a mutated N-terminal exon 1 HTT fragment [17]. The *Hdh*Q150 mice have an expanded CAG repeat knocked in to the mouse huntingtin gene (*Htt*) [18],[19], which is partially mis-spliced with the result that these mice express mutant versions of both an exon 1 HTT and a full-length HTT protein [20]. Because *Hdac4* knock-out (*Hdac*4KO) mice die in early postnatal life [21], the HD mutation could not be transferred onto an *Hdac4* null background. Therefore, we crossed males for each of the HD mouse models to *Hdac4*HET females (Figure 1A). Analysis of the progeny indicated that *Hdac4* mRNA levels were decreased to approximately 50% in both *Hdac4*HET and double-mutant mice in both crosses (Figure 1B).

Since HDAC4 functions as a transcriptional corepressor [22], it was important to check whether *Hdac4* knock-down regulated the expression of the R6/2 transgene, as this would be expected to modulate the onset and progression of disease in R6/2 mice. Therefore, we used Taqman qPCR to demonstrate that exon 1 *HTT* mRNA was not altered in the cortex (Figure 1C), cerebellum, nor striatum (Figure S1A) of Dble::R6/2 mice. Similarly, we showed that endogenous *Htt* levels were unchanged as a consequence of HDAC4 reduction in both R6/2 and *Hdh*Q150 mice (Figures 1D and S1B). In addition, given that CAG repeat length is linked to aggregation kinetics and disease progression, we ensured that the CAG repeats were maintained at comparable levels throughout the course of this study (Table S1).

Alteration of HDAC4 levels has been shown to modulate *Hdac9* in muscle cells [23] and *Hdac5* in primary mouse hepatocytes [24]. Therefore, we used Taqman qPCR to show that *Hdac4* knock-down did not affect the levels of the other 10 *Hdacs* in brain regions of mice that did or did not express the HD mutation (Figures 1E and S1C and S1D).

### HDAC4 Knock-Down Reduces Aggregate Load and Increases Levels of Soluble HTT in Mouse Models of HD

Bioinformatic predictions of HDAC4 structure suggested that HDAC4 has an N-terminal coil–coil domain within which it possesses short polyQ tracts that might convey an increased propensity for amyloid formation [15] as was confirmed in cultured cells [13]. Hence, we hypothesized that HDAC4 might exhibit pro-aggregation properties in HD mouse models. In order to investigate the molecular consequences of HDAC4 knock-down in HD mice, we employed the Seprion ligand ELISA to quantify aggregate load [25] and time-resolved Förster resonance energy transfer (TR-FRET) to measure soluble mutant HTT levels [26]. TR-FRET detects a FRET signal between two appropriately labelled antibodies. In this case, 2B7 (epitope: 1–17 amino acids of HTT) is paired with MW1 (epitope: polyQ in nonaggregated HTT).

**Figure 2:**
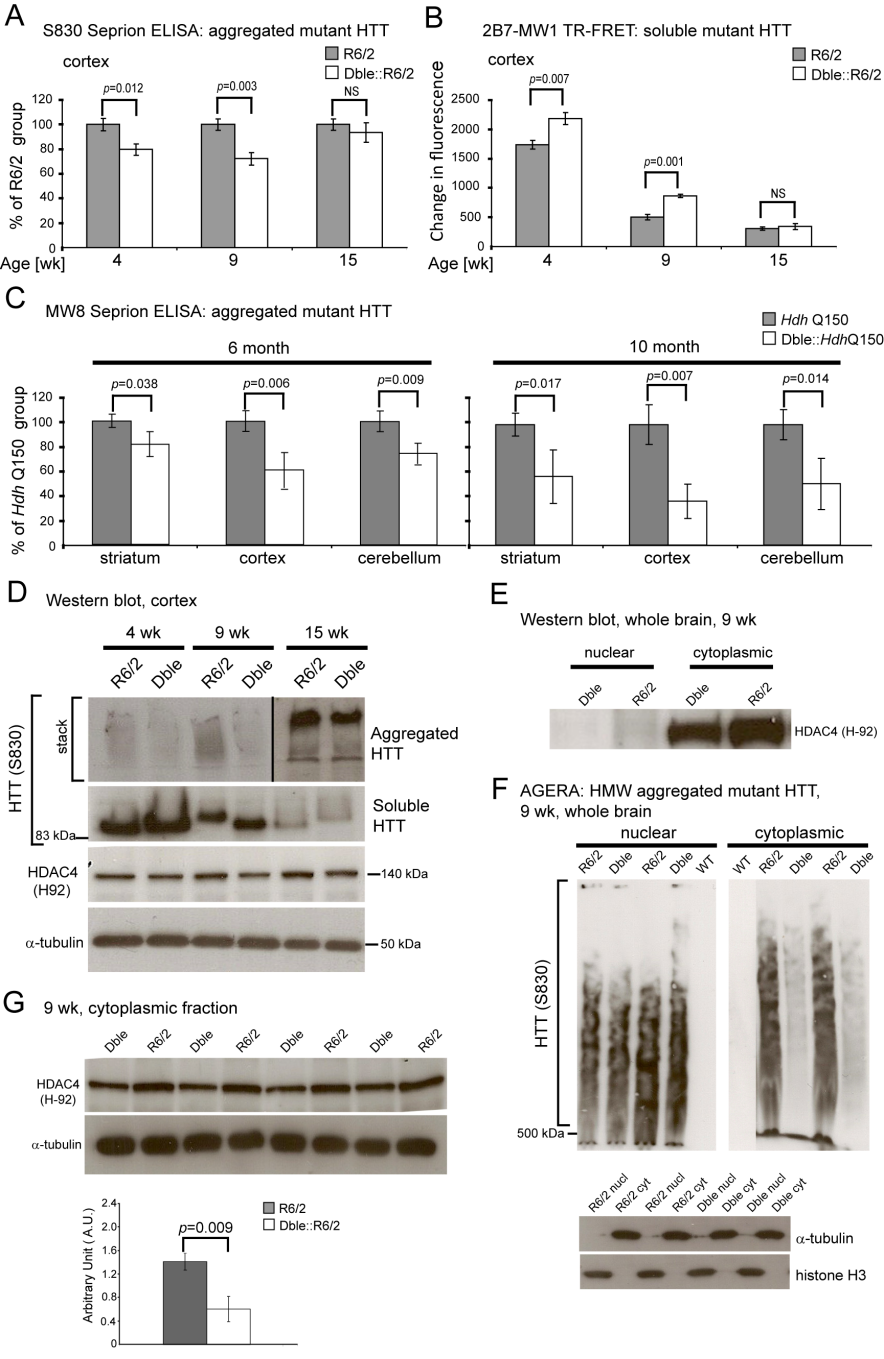
HDAC4 knock-down delays aggregate formation in R6/2 and *Hdh*Q150 mouse models of HD. (A) Seprion ligand ELISA was used to quantify aggregate load in the cortex of R6/2 and Dble::R6/2 mice at 4, 9, and 15 wk of age. Values for the Dble::R6/2 mice were plotted as a percentage of R6/2 aggregate load (*n* = 6). (B) TR-FRET was used to determine the levels of soluble exon 1 HTT in the cortex of R6/2 and Dble::R6/2 mice at 4, 9, and 15 wk of age (*n* = 6). (C) Seprion ligand ELISA was used to quantify aggregate load in the striatum, cortex, and cerebellum of *Hdh*Q150 and Dble::*Hdh*Q150 mice at 6 and 10 mo of age. Values for the Dble::*Hdh*Q150 mice were plotted as a percentage of aggregate load of *Hdh*Q150 mice (*n*≥7). (D) Representative S830 immunoblot of cortical lysates showing the difference in soluble and aggregated exon 1 HTT between R6/2 and Dble::R6/2 (Dble) mice and how this change occurs with age. (E) Comparison of HDAC4 levels in the nuclear and cytoplasmic fractions of R6/2 and Dble::R6/2 (Dble) brains by western blot. The purity of the fractions is shown in Figure S2D. (F) Western blot of detergent-insoluble high molecular weight (HMW) aggregates isolated from the nuclear and cytoplasmic fractions of R6/2 and Dble::R6/2 (Dble) brains, resolved by agarose gel electrophoresis (AGERA), and immunodetected with the S830 antibody (representative of three experiments) (*n* = 8). The purity of the fractions is shown by western blotting with α-tubulin and histone H3. (G) Western blot of HDAC4 in the cytoplasmic fraction of R6/2 and Dble::R6/2 (Dble) brains at 9 wk of age. HDAC4 levels were measured by densitometry and calculated relative to α-tubulin. Error bars are SEM. *p* values were calculated using Student's *t* test.

In R6/2 mice, the level of soluble exon 1 HTT decreases with disease progression as a consequence of its aggregation. The Seprion ELISA revealed that HDAC4 knock-down reduced the aggregate load in the cortex (Figure 2A), brain stem, hippocampus, and cerebellum (Figure S2A) of Dble::R6/2 mice at 4 and 9 wk but that this effect had diminished by 15 wk of age. Accordingly, TR-FRET demonstrated that reduced HDAC4 levels led to an increase in soluble exon 1 HTT in the cortex (Figure 2B), brain stem, hippocampus, and cerebellum (Figure S2B) of Dble::R6/2 mice at 4 and 9 but not at 15 wk of age. This shift in the ratio between soluble and aggregated exon 1 HTT levels can be visualised on the western blots in Figure 2D. We performed Seprion ELISA to determine whether similar results could be obtained in the *Hdh*Q150 knock-in mice. A significant reduction in the aggregate load was observed in the striatum, cortex and cerebellum of Dble::*Hdh*Q150 mice at 6 and 10 mo of age (Figure 2C). Taken together, these data show that HDAC4 knock-down significantly reduced aggregate load and increased levels of soluble mutant HTT in HD mouse models, reflecting a delay in the aggregation process.

We used *Hdac4*KO P3 brain tissue to confirm that the commercially available antibodies Sigma (DM-15), Santa Cruz (H-92), and Cell Signalling (CS2072) detected an HDAC4 specific signal (Figure S2C and unpublished data). On western blotting of nuclear and cytoplasmic fractions of mouse brain, we found that only trace amounts of HDAC4 could be detected in the nuclear fraction (Figure 2E). Therefore, to investigate whether the reduction in aggregation occurred in the cytoplasm, as would be consistent with the presence of HDAC4, we perfomed cellular fractionation on 4 wk and 9 wk brain tissue. We then resolved detergent insoluble high-molecular weight aggregates from the nuclear and cytoplasmic lysates by agarose gel electrophoresis (AGERA), prepared western blots, and performed immunodetection with the S830 antibody (epitope: exon 1 HTT). We found that HDAC4 knock-down reduced the aggregate load in the cytoplasm but not in the nucleus of Dble::R6/2 mice at both 4 (Figure S2D) and 9 (Figure 2F) wk of age. Consistent with this, we found that the cytoplasmic steady-state levels of HDAC4 were significantly reduced in Dble::R6/2 mice as compared to R6/2 at both 4 (Figure S2E) and 9 (Figure 2G) wk of age. The purity of the cellular fractions was validated by immunoblotting with α-tubulin and histone H3 antibodies (Figures 2F and S2D).

### The Decrease in Aggregate Load Is Not a Consequence of Strain Background

The R6/2 colony was maintained by backrossing to (CBA/Ca×C57BL/6J)F1 mice and the *Hdac4* knock-out mice had been bred to the same F1 background for more than six generations. We know from having bred R6/2 mice for 99 generations and from multiple experiments that the differential segregation of CBA/Ca and C57BL/6J alleles has no effect on HD-related phenotypes in R6/2 mice [10],[27]–[33]. However, the *Hdac4* null allele had been created on a 129S mouse strain background, and it is inevitable that even after backcrossing to (CBA/Ca×C57BL/6J)F1 mice for multiple generations, the *Hdac4* null allele would be retained in a genomic region of 129S DNA that had not been removed by recombination. Therefore, it was possible that the observed effects might be due to genetic variation in the 129S *Hdac4*-linked haplotype rather than through a reduction in HDAC4. To rule out this scenario, we identified an *Hdac4*-linked single nucleotide polymorphism (SNP) that was polymorphic between 129S and both the C57BL/6J and CBA/Ca strain backgrounds. We crossed R6/2 mice to (129S×C57BL/6J)F1s and identified R6/2 progeny that either did not carry or were heterozygous for the 129S SNP (*n* = 7/genotype). The heterozygous mice contained the 129S haplotype with a wild-type *Hdac4* allele. Seprion ELISA on 9-wk-old cortex showed that the 129S *Hdac4*-linked haplotype did not modify aggregate load in R6/2 mice (Figure S2F), confirming the role for HDAC4 in aggregate reduction.

### HDAC4 Associates with Mutant HTT and Colocalizes with Cytoplasmic Inclusions

**Figure 3:**
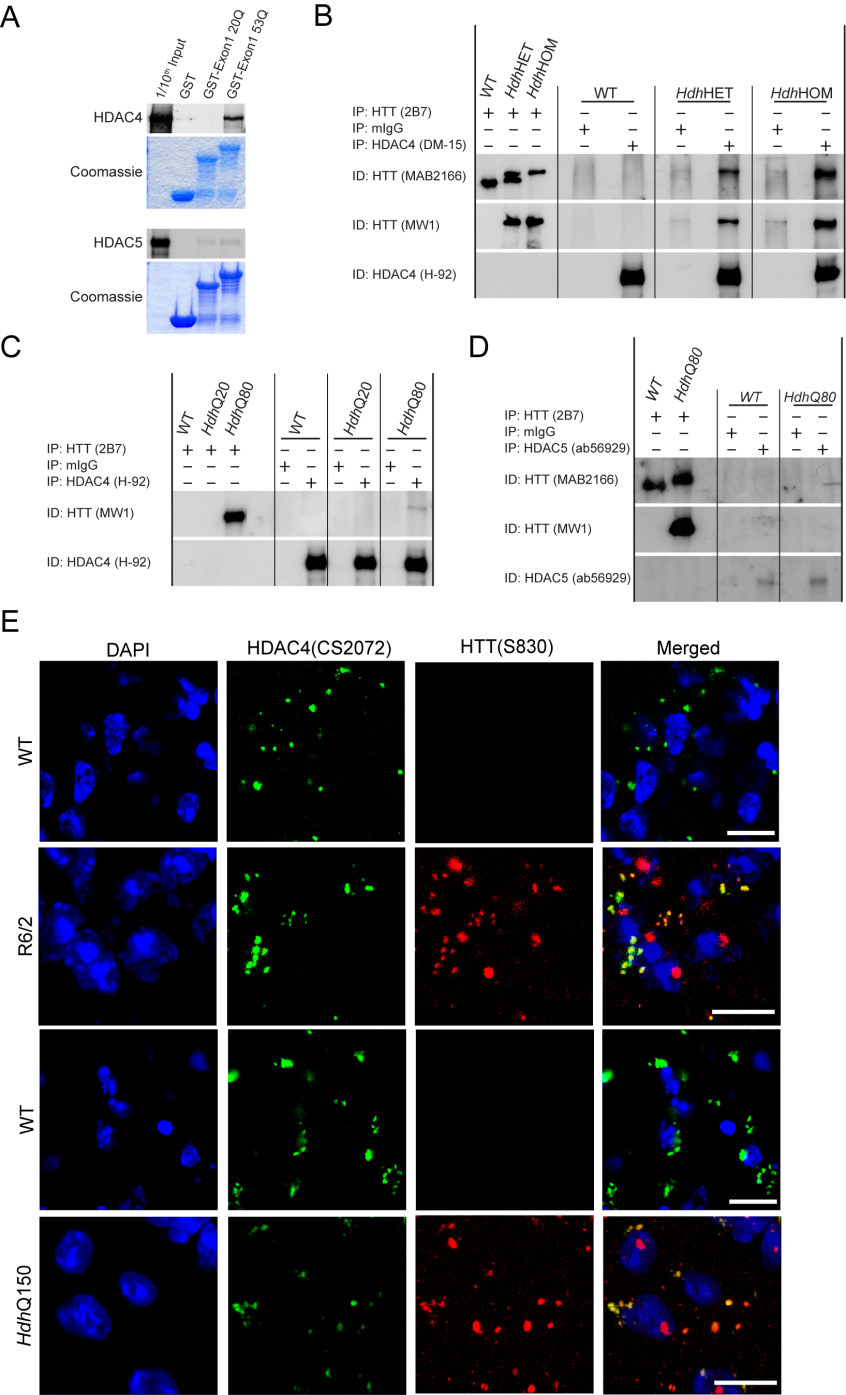
HDAC4 interacts with mutant huntingtin *in vitro* and *in vivo* and colocalizes with cytoplasmic inclusions. (A) GST pull-down assays revealed that HDAC4 interacts with mutant (53Q) but not WT (20Q) exon 1 HTT. In contrast HDAC5 weakly interacts with both mutant and WT exon 1 HTT. The coomassie stained gel shows the exon 1 HTT GST fusion proteins that were used to pull-down ^35^S-methioine labelled recombinant HDAC4 or HDAC5. (B) Western blot probed for HTT (MAB2166 or MW1) after immunoprecipitation with HDAC4 (DM-15) from brain tissue from 8-wk-old WT and *Hdh*Q150 heterozygous and homozygous mice (representative picture of three independent experiments). (C) Western blot probed for mutant HTT (MW1) after immunoprecipitation with HDAC4 (H-92) from brain tissue from 8-wk-old WT, *Hdh*Q20, and *Hdh*Q80 homozygous mice. (D) Western blot probed for mutant HTT (MAB2166 or MW1) after immunoprecipitation with HDAC5 (ab56929) from brain tissue from 8-wk-old WT and *Hdh*Q80 homozygous mice. (E) Representative immunofluorescence images of cortex from 14-wk-old R6/2 and 23-mo-old *Hdh*Q150 mice immunostained for mutant HTT (S830) and HDAC4 (CS2072) and counterstained with DAPI. A similar pattern of cytoplasmic co-localisation was also seen in the striatum and hippocampus. Scale bar, 15 µm. IP, immunoprecipitation; ID, immunodetection.

To further understand the nature of the reduction in aggregate load by HDAC4 knock-down, we reasoned that HDAC4 might associate with HTT. Hence, we employed an *in vitro* GST pull-down assay and found that HDAC4 interacted specifically with exon 1 HTT containing a 53 polyQ tract but not with its 20 polyQ counterpart (Figure 3A). To determine whether HDAC4 associates with endogenous HTT, we immunoprecipitated HTT (2B7, epitope 1–17) or HDAC4 (DM-15) from brain lysates of 8-wk-old WT (7Q), *Hdh*Q150 heterozygous, and *Hdh*Q150 homozygous mice and immunoblotted with the MW1 (soluble mutant HTT), MAB2166 (soluble wild type and mutant HTT), and H-92 (HDAC4) antibodies. We found that mutant but not wild-type HTT co-immunoprecipitated with HDAC4 (Figure 3B). To investigate the effect of polyQ length on this interaction, we repeated the experiment with lysates from 8-wk-old heterozygous knock-in mice carrying polyQ tracts of 20 (*Hdh*Q20) or 80 (*Hdh*Q80). We found that HDAC4 could immunoprecipitate HTT containing 80 but not 20 glutamines (Figure 3C), consistent with the *in vitro* pull-down data. The sequence of HDAC4 is very similar to the class IIa member HDAC5. Therefore, to investigate the specificity of these interactions, we repeated the *in vitro* and *in vivo* immunprecipitations with an antibody specific to HDAC5. Although there was a weak interaction between exon 1 HTT and HDAC5 by *in vitro* pull-down (Figure 3A), this was not specific to mutant HTT, as was the case for HDAC4, and HDAC5 failed to immunoprecipitate HTT from brain lysates (Figure 3D).

To further explore this association between HDAC4 and mutant HTT, we performed immunohistochemistry to determine whether HDAC4 co-localized with nuclear and/or cytoplasmic inclusions. For this purpose, we validated a number of commercially available antibodies and found CS2072 to be specific for HDAC4 by fluorescent immunolabelling as it gave no signal on HDAC4KO P3 brain sections (Figure S3A). Consistent with our western blot results, HDAC4 was localised to the cytoplasm appearing as a punctate pattern in adult brains (Figures 3E and S3B and S3C). This cytoplasmic localisation of HDAC4 is supported by its co-localisation with the synaptic markers synaptophysin and PSD95 (Figure S3C). Confocal microscopy showed that the S830 HTT antibody detects huntingtin aggregates in R6/2 and *Hdh*Q150 brains and that HDAC4 co-localised with some but not all cytoplasmic inclusions (Figures 3E and S3B) in both cases. Taken together, our data indicate that HDAC4 associates with soluble mutant HTT *in vivo* and co-localizes with cytoplasmic inclusions in all brain regions studied.

### HDAC4 Reduction Does Not Rescue Global Transcription Dysregulation

**Figure 4:**
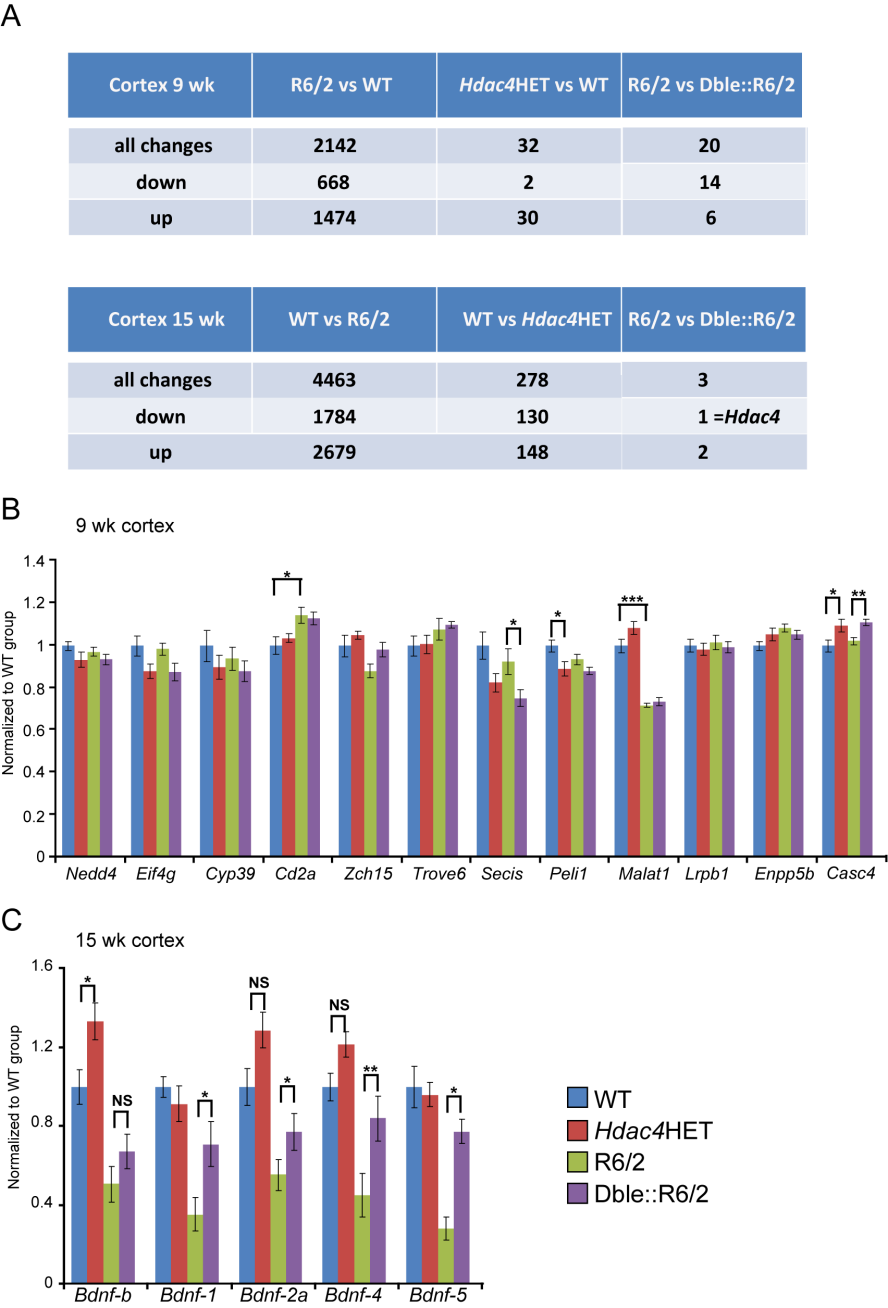
HDAC4 knock-down does not rescue global transcriptional dysregulation. (A) Affymetrix arrays were used to determine the effect of *Hdac4* knock-down on the cortical transcription profile of WT and R6/2 mice at 9 and 15 wk of age (*n*≥8 per genotype per time point). The number of genes that were significantly altered between genotypes with a fold-change of >30% for each pairwise comparison is noted. Statistical significance was determined after FDR-correction at a stringency of *p*≤0.05. (B) Taqman qPCR validation of the genes that were predicted to be differentially expressed between R6/2 and Dble::R6/2 cortex at 9 wk of age. See Table S3 for gene abbreviation definitions. (C) Cortical *Bdnf* mRNA levels for promoter transcripts 1, 2a, 4, and 5 as well as the coding exon (B) were assessed by Taqman qPCR at 15 wk of age. All Taqman qPCR values were normalized to the geometric mean of three housekeeping genes: *Atp5b*, *Canx*, and *Rpl13a*. Error bars are S.E.M (*n* = 8). *p<0.05, **p<0.01, ***p<0.001; NS, not significant.

Transcriptional dysregulation is a well-documented molecular characteristic of HD pathogenesis. A comparative study of the striatal transcription profiles of seven mouse models and HD *post mortem* brains showed that the dysregulated signature in R6/2 and *Hdh*Q150 models was highy comparable and in both cases more closely replicated that observed in human HD tissue than that of the other mouse models [34]. HDAC inhibitors were initially pursued as a therapy for HD because of their potential for reversing these transcriptional changes. Therefore, we performed Affymetrix microarray profiling of 9- and 15-wk cortex for WT, *Hdac4*Het, R6/2, and Dble::R6/2 mice to assess whether HDAC4 knock-down might rescue the global transcriptional dysregulation that occurs in R6/2 mice. As expected the cortical expression profile was profoundly changed between WT and R6/2 mice by 9 wk of age and was further dysregulated at 15 wk (Figure 4A). However, comparison of the R6/2 and Dble::R6/2 profiles indicated that only a very small number of probe sets were predicted to be differentially expressed at statistically significant levels (Figure 4A). This suggested that reduction in HDAC4 had not served to rescue transcriptional dyregulation. We used Taqman qPCR to validate the predicted changes in gene expression between R6/2 and Dble::R6/2 cortex. We were only able to detect statistically different expression levels of small effect size for *Secis* and *Casc4*, and in both cases this was in the opposite direction of that predicted by the arrays (Figure 4B).

Dysregulation of *Bdnf* promoter transcripts is a well-characterised hallmark of disease progression in HD [35], and restoration of *Bdnf* levels has been shown to correlate with phenotypic improvements in HD mouse models. As *Bdnf* probe sets were not represented on the arrays, we used Taqman qPCR to measure the levels of multiple *Bdnf* promoter trancripts as well as the coding exon (*Bdnf-b*) in cortex at 15 wk of age. We found that HDAC4 reduction increased *Bdnf-b* levels in WT cortex and almost restored the R6/2 dysregulated *Bdnf* transcripts to WT levels in Dble::R6/2 mice (Figure 4C).

### HDAC4 Knock-Down Improves MSN Membrane Properties and Cortico-Striatal Synaptic Function

**Figure 5:**
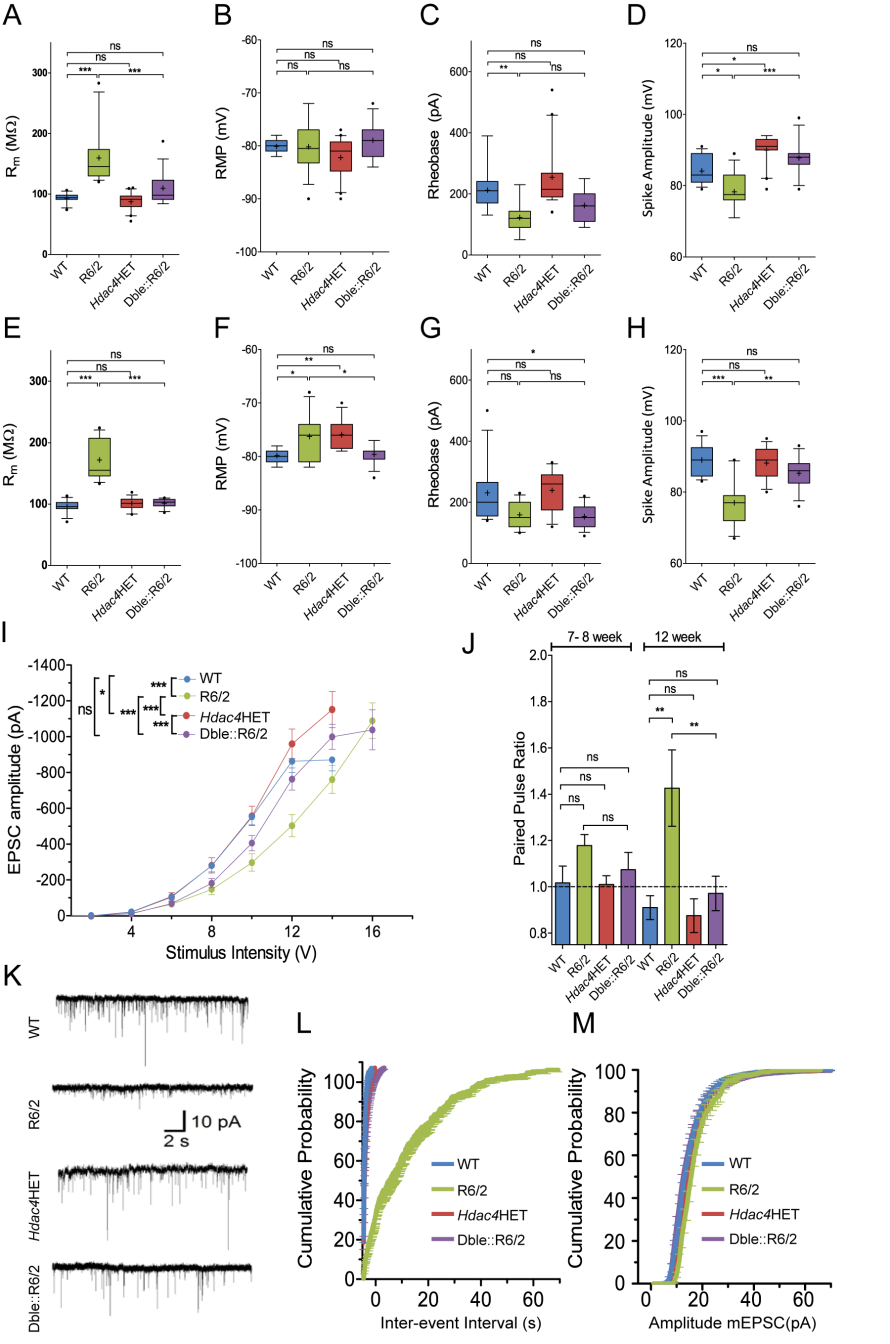
HDAC4 reduction improves the electrophysiological characteristics of MSNs and corticostriatal synaptic function. (A–H) Box and whisker plots of (A, E) input resistance (R_m_), (B, F) RMP, (C, G) rheobase current, and (D, H) spike amplitude from corticostriatal slices at (A–D) 7–8 and (E–H) 12 wk of age. Box and whisker plots: +, mean; box, interquartile range; whisker, 10–90 percentile; outliers, closed circles. (I) Measurement of evoked EPSCs following cortical stimulation showed that the R6/2 mice show lower basal transmission compared to WT or *Hdac4*HET and that this is restored in the Dble::R6/2 mice at 7–12 wk of age. (J) R6/2 MSNs have a higher paired-pulse ratio than WT (interstimulus interval, 20 ms), indicating reduced glutamate release probability. This is fully restored in Dble::R6/2 mice at 12 wk of age. (K) Representative traces of miniature EPSCs (mEPSCs) at 8 wk. R6/2 mice show strongly depressed mEPSC frequency, which is significantly rescued in the Dble::R6/2 mice. (L, M) Average cumulative plot of mEPSC interevent interval (0.1 s bins) (L) or amplitude (1 pA bins) (M); *n* = 11–12 for all four genotypes. Statistical analysis was performed by (A–H and J) one-way ANOVA with Tukey's multiple comparison test, (I) two-way ANOVA with Bonferroni multiple comparison test, and (L, M) Kolmogorov–Smirnov (KS) test. *p<0.05, **p<0.01, ***p<0.001.

MSNs in symptomatic R6/2 mice show pronounced morphological abnormalities, including dendritic shrinkage and spine loss. Largely consistent with these anatomical changes, at a behaviorally symptomatic age, R6/2 MSNs display a marked increase in membrane resistance, depolarization of the resting membrane potential (RMP), and an increased intrinsic excitability in response to current injection [36]. These phenotypes indicate that the R6/2 MSNs are hyperexcitable relative to MSNs in WT animals. In addition, symptomatic R6/2 mice display a progressive impairment in corticostriatal connectivity [37]. In combination, this reduction of cortical input, coupled with the abnormal excitability of the MSNs within the R6/2 striatum, will have serious consequences for appropriate striatal information processing and resultant basal ganglia output. These features likely contribute and in part underlie the impaired behavioral function exhibited by these mice. We determined the extent of functional improvement in MSNs from Dble::R6/2 as compared to R6/2 mice at 7–8 and 12 wk of age. As previously published, R6/2 MSNs exhibited a higher membrane resistance than those from WT and *Hdac4*HET mice at both ages, which was restored to WT levels in Dble::R6/2 mice (Figure 5A,E).

While R6/2 MSNs at 7–8 wk of age were not significantly depolarized (Figure 5B), by 12 wk the RMP was depolarized by approximately 3 mV relative to WT MSNs, and this was rescued in the Dble::R6/2 mice (Figure 5F). Despite previous reports of reduced rheobasic current (minimum current injection required to elicit an action potential) in R6/2 mice [36], this was a modest phenomenon in our hands and the reduction of HDAC4 in the Dble::R6/2 mice had no effect (Figure 5C,G). Action potential amplitude was, however, significantly reduced in R6/2 compared to WT and *Hdac4*HET at both ages and was fully restored in the Dble::R6/2 mice (Figure 5D,H). To assess an improvement in corticostriatal transmission, glutamatergic excitatory postsynaptic currents (EPSCs) were evoked by stimulating layer V cortical afferents innervating the striatum. In both 7–8 and 12 wk age groups, R6/2 mice showed significant reduction in EPSC amplitude for any given stimulus intensity applied. There was no difference in evoked EPSCs per genotype between the two age groups, and the data were therefore pooled (*n* = 28–32 neurons per genotype; Figure 5I). In the Dble::R6/2 mice, a significant restoration of evoked EPSC amplitude compared to R6/2 was noted. To further delineate the locus of this improvement, a paired-pulse stimulation paradigm (20 ms interstimulus interval) was employed to specifically assess glutamate release probability. R6/2 corticostriatal synapses displayed higher paired-pulse ratios than WT synapses, and this was significant in the 12-wk dataset, indicative of impaired glutamate release in this age group. This was fully restored in the Dble::R6/2 mice (Figure 5J). In agreement with the reduction in evoked glutamate release in R6/2 corticostriatal synapses, quantal glutamate release within the striatum, which can arise from release at both thalamostriatal and corticostriatal presynaptic terminals, was severely depressed in the 7–8-wk-old mice [mean frequency of mEPSCs in MSNs from WT = 2.3±0.25 Hz (*n* = 12), *Hdac4*HET = 1.87±0.38 Hz (*n* = 11), R6/2 mice = 0.13±0.03 Hz (*n* = 11)]. The Dble::R6/2 mice showed a profound rescue in this phenotype, with a mean frequency of mEPSCs of 1.1±0.14 Hz (*n* = 12) (significance against R6/2 frequency assessed by Kolmogirov–Smirnoff analysis *p*<0.0001; Figure 5K,L). There was no change in the amplitude of mEPSCs in R6/2 compared to WT mice, suggesting that postsynaptic 2-amino-3-(5-methyl-3-oxo-1,2-oxazole-4-yl) propanoic acid (AMPA) receptor function was not impaired in the HD model, and did not contribute to the impairment in glutamatergic transmission. mEPSC amplitude was unchanged in the *Hdac4*HET or Dble:R6/2 animals relative to WT or R6/2 mice (Figure 5M).

In conclusion, the combined restoration of the membrane properties of MSNs from the Dble:R6/2 mice alongside the improvement in glutamatergic cortical input to the striatum would be expected to significantly improve striatal information processing, normalize aberrant basal ganglia output, and result in an improvement in behavioral function.

### HDAC4 Knock-Down Improves Neurological Phenotypes in R6/2 Mice

In order to evaluate whether the molecular and electrophysiological changes that had been detected in the Dble::R6/2 mice might ameliorate HD-related behavioural and physiological phenotypes, we employed a set of quantitative, well-characterised tests. Mice were well matched for CAG repeat length (Table S1), and phenotypic parameters were measured from 4 to 15 wk of age in WT, *Hdac4*HET, R6/2, and Dble::R6/2 mice. All analyses were performed blind to genotype, and in all cases, the progression of R6/2 phenotypes was consistent with previous reports.

**Figure 6:**
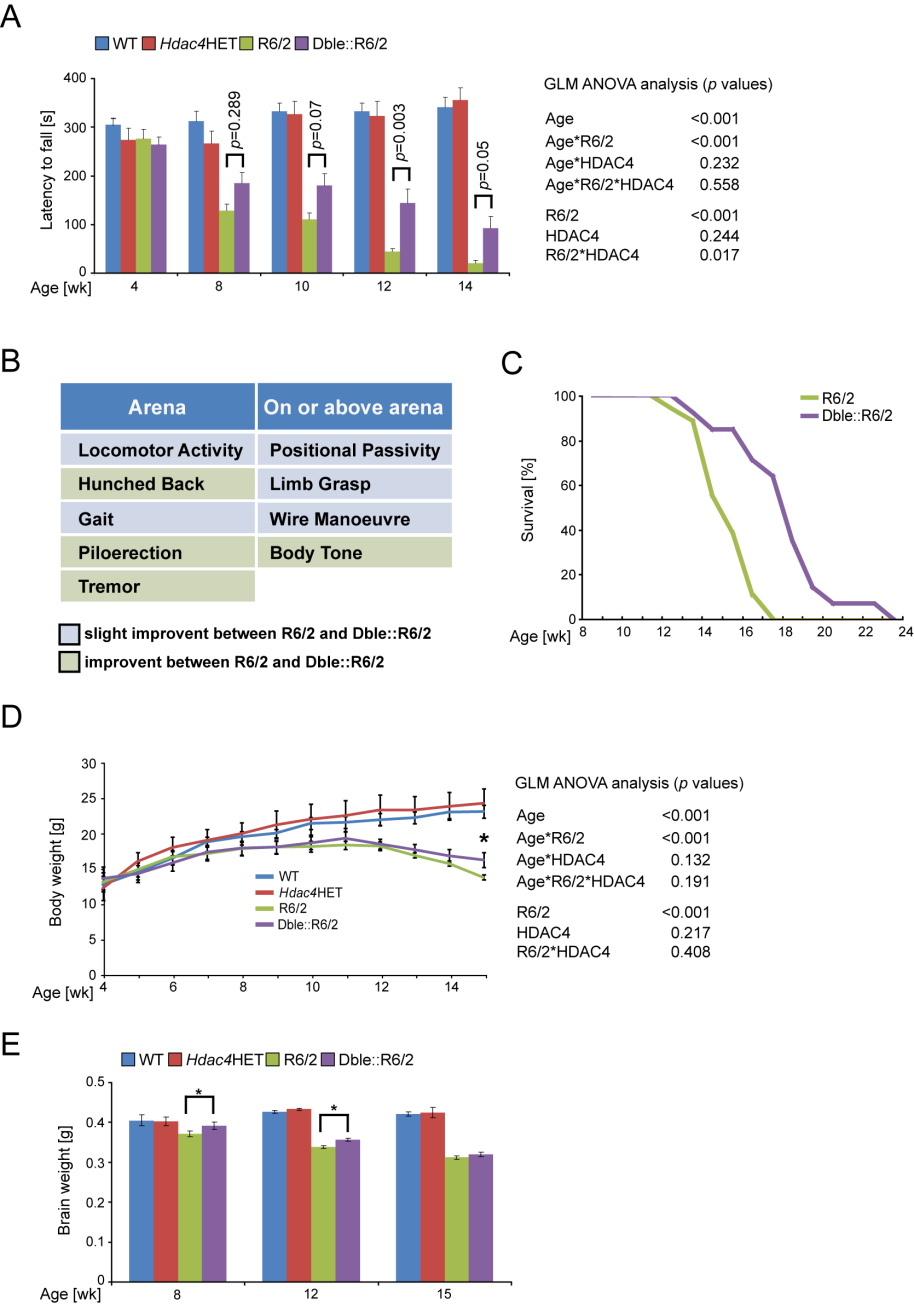
HDAC4 knock-down improves neurological phenotypes and extends survival. (A) Reduction in HDAC4 results in a pronounced delay in impaired motor coordination as determined by rotarod performance (*n*≥13/genotype). (B) Assessment of neurological phenotypes via a modified SHIRPA protocol (see Table S2 for details). Of the nine parameters that distinguished R6/2 and WT mice, all were improved in the Dble::R6/2 mice. See also Video S1 and Video S2. (C) Kaplan–Meier analysis showed that knock-down of HDAC4 significantly increased R6/2 survival (*n*≥13/genotype). (D) Reduction of HDAC4 did not result in an overall improvement in the failure of R6/2 mice to gain weight, although Dble::R6/2 mice were significantly heavier than R6/2 mice at 15 wk of age (*n*≥13/genotype). (E) There was a slight but statistically significant increase in the brain weight of Dble::R6/2 as compared to R6/2 mice at 4 and 9 wk of age (*n*≥10/genotype). Statistical analysis was performed by GLM-ANOVA with Greenhouse Geisser *post hoc* analysis (B and D), by multiple comparisons using Bonferroni post hoc test (B and D), and by log-rank test (C).

Rotarod performance is a sensitive indicator of balance and motor coordination that has been reliably shown to decline in R6/2 mice. In line with previous results, R6/2 rotarod performance was impaired by 8 wk (*p*<0.001) and deteriorated further with age (Figure 6A). HDAC4 knock-down had no impact on the performance of WT mice. However, it significantly improved R6/2 rotarod performance at all ages and delayed the progression of rotarod impairment by 1 mo to the extent that 12-wk-old Dble::R6/2 mice performed as well as 8-wk-old R6/2 mice (Figure 6A).

At 14 wk of age, close to end stage disease, the deterioration in the appearance of the Dble::R6/2 mice was considerably less marked than that of R6/2. To document this, we performed a modified SHIRPA analysis [38] and measured 16 phenotypic parameters (Table S2), for which nine gave a positive score in R6/2 as compared to WT mice. In all cases, the appearance of these phenotyes was improved in the Dble::R6/2 mice when compared to R6/2 (Figure 6B). In particular, piloerection and tremor could not be detected in Dble::R6/2 mice, and hunched back and body tone were vastly improved (Figure 6B). The phenotypic improvements are evident in Videos S1 and S2, and in the light of these, it was surprising that we were unabe to detect an any marked decrease in body weight loss (Figure 6D). The dramatic improvement in the appearance of the mice led us to assess whether HDAC4 knock-down had pro-survival effects, and we found that it extended the lifespan of R6/2 mice by approximately 20%, *p* = 0.0004 (log-rank test) (Figure 6C).

Brain weight was measured for cohorts of mice that were culled at 8, 12, and 15 wk of age, and as previously described, there was a progressive decrease in the weight of R6/2 brains as compared to WT. HDAC4 knock-down resulted in a very slight but statistically significant increase in brain weight at 9 and 12 but not at 15 wk of age (Figure 6E).

## Discussion

There is mounting support for the use of HDAC inhibitors in the treatment of a wide range of brain disorders, predominantly aimed at pathological alterations in the brain transcriptome. HDAC4 is a transcriptional repressor that is normally retained in the cytoplasm, but localises to the nucleus upon de-phosphorylation [39],[40]. In line with previous reports [41], we found that HDAC4 was located in the cytoplasm in mouse brain and showed that it did not relocate to the nucleus during disease progression. We demonstrated that HDAC4 associates with mutant HTT in a polyQ-dependent manner *in vivo*, consistent with an association that occurs between the polyQ stretch in HTT and the Q-rich domain of HDAC4. In line with these observations, we found that HDAC4 co-localised with cytoplasmic inclusions in both R6/2 and knock-in HD mouse models. The genetic knock-down of HDAC4 led to a significant delay in the cytoplasmic aggregation process and led to a significant restoration of synaptic function. At a physiological level, knock-down of HDAC4 extended lifespan and partially restored motor coordination and other neurological phenotypes. This suggests that a cytoplasm-based pathophysiological mechanism contributes to key aspects of neurodegenerative phenotype observed in HD.

A cytoplasmic mechanism of action for HDAC4-mediated beneficial effects was unexpected. In general, HDACs have been pursued as therapeutic targets because of their impact on epigenetics and transcription. HDAC4 is known to repress *MEF2* in muscle [40], and it has been proposed that HDAC4 binds to HDAC3 to activate its deacetylase domain [24]. However, we found no impact on global transcriptional dysregulation upon HDAC4 knock-down in R6/2 mice, consistent with a predominantly cytoplasmic localisation of HDAC4. Surprisingly, the absence of HDAC4 in knock-out postnatal brain tissue had little effect on the brain transcriptome [42]. In fact, it has been shown that HDAC4 does not function as a lysine-deacetylase [43], and consistent with this, we found that HDAC4 knock-out had no effect on global acetylation in brain *in vivo* [42]. Our demonstration that the dysregulation of *Bdnf* promoter transcripts was alleviated in the double mutant mice is consistent with a cytoplasmic-based mechanism of action. *Bdnf* expression is repressed by RE1 silencing transcription factor (REST), which is retained in the cytoplasm in a complex containing HTT and which translocates to the nucleus in response to HD pathology [44]. The cytoplasmic aggregation process initiates prior to a reduction in *Bdnf* transcripts in R6/2 and other HD mouse models, and therefore it is not surprising that a delay in this cytoplasmic pathology might in turn result in a delay in *Bdnf* dysregulation.

Our demonstration that a delay in the cytoplasmic aggregation process has beneficial consequences is supported by the published correlation between the appearance of neuropil aggregates and disease progression [45] and is consistent with previous predictions [46]. In the human HD *post mortem* brain, neuropil aggregates are much more common than nuclear inclusions, present in a large numbers, and potentially associated with onset of clinical symptoms [47]. Our data suggest that HDAC4 might modulate HTT aggregation through a direct interaction serving to template or nucleate soluble HTT. Alternatively, given that HDAC4 may self-aggregate, it could influence HTT aggregation indirectly through perturbation of proteostasis networks [48]. The delay in HTT aggregation afforded by a reduction in HDAC4 was most pronounced in presymptomatic and early-stage disease and diminished with disease progression, presumably reflecting changes in aggregation kinetics. This association between phenotypic improvements and a shift from aggregated to soluble mutant exon 1 HTT is consistent with our previously published *in vivo* studies [49],[50]. Our data provide no information as to the aggregate species that is toxic, or as to whether all species of aggregates have detrimental consequences, but only indicate that shifting the equilibrium toward soluble HTT is beneficial *in vivo*.

The improvement in synaptic function as a consequence of HDAC4 knock-down was not related to a restoration in the expression level of dysregulated synaptic transcripts. Instead, it may act through reducing cytoplasmic aggregation as neuropil aggregates have been shown to inhibit axonal transport, synaptic function, and glutamate release in HD fly models [51]. The restoration of corticostriatal synaptic function demonstrated that the reduction of HDAC4 has functional consequences in the brain. However, in this study, HDAC4 was ubiquitously knocked down, and as HD has a peripheral component to its pathophysiology [52], it is conceivable that the reduction of HDAC4 also had beneficial consequences in tissues other than the brain. Given that HDAC4 has well-established functions in muscle, that muscle atrophy is a major symptom of HD, and that HDAC4 has been linked to disease progression in an ALS mouse model [53], we are currently investigating the extent to which HDAC4 reduction in muscle might contribute to the improved HD phenotypes.

The administration of HDAC inhibitors has been shown to improve disease phenotypes in a wide range of HD models [5]. To better understand which HDACs, when inhibited, are most responsible for these beneficial consequences, we embarked on a series of genetic manipulations in HD mouse models. In this article we show that the genetic knock-down of HDAC4 delayed cytoplasmic aggregation, improved synaptic function, and improved disease phenotypes. In contrast, the genetic knock-down of HDAC3 [27] and the class IIa members HDAC7 [10], HDAC5, and HDAC9 (our unpublished data) had no effect on R6/2 phenotypes. Strikingly, we recently showed that administration of SAHA caused a reduction in HDAC2 and HDAC4 at the protein but not RNA level in some R6/2 brain regions and that this correlated with a reduction in aggregation and a restoration of cortical *Bdnf* transcripts [11]. Therefore, we speculate that the beneficial effects of SAHA were at least in part transmitted through the down-regulation of HDAC4 via a mechanism not related to its enzyme activity.

Perhaps the best validated therapeutic target for HD is the HTT protein, and the reduction of HTT through gene silencing is being developed using both antisense oligonucleotides and RNAi [54]–[56]. However, delivery to the brain is a major challenge for these approaches, and as HTT has many essential functions, the potential liability of decreasing HTT to a detrimental level cannot be ignored. We have shown that reduction of HDAC4 shifts the ratio from aggregated to soluble HTT and therefore acts directly on the HD mutation. Our demonstration that HDAC4 levels can be decreased through the administration of a small brain-penetrant molecule (SAHA) is extremely promising as more selective inhibitors (e.g., specific to HDAC4 or class IIa enzymes) may have similar effects, making it possible to target HTT aggregation with a small molecule. Finally, these findings may have wider implications as HDAC4 is a component of Lewy Bodies in Parkinson's disease brains [57] and administration of SAHA improved the synaptic plasticity and learning behaviour in an Alzheimer disease model [58].

## Materials and Methods

### Ethics Statement

All animal work was approved by the local ethics committees and was performed in accordance with UK Home Office regulations or the Swiss Law (Kantonales Veterinäramt Basel-Stadt).

### Mouse Maintenance and Breeding, Genotyping, and CAG Repeat Sizing

Hemizygous R6/2 mice were bred by backcrossing R6/2 males to (CBA×C57BL/6)F1 females (B6CBAF1/OlaHsd, Harlan Olac, Bicester, UK). Similarly, the *Hdac4* knock-out colony [21] was maintained by backcrossing heterozygous males to B6CBAF1/OlaHsd females. *Hdh*Q150 homozygous mice on a (CBA×C57BL/6)F1 background were obtained by intercrossing *Hdh*Q150 heterozygous CBA/Ca and C57BL/6J congenic lines as described previously [19]. The *Hdh*Q20 and *Hdh*Q80 mice were from CHDI colonies at The Jackson Laboratory (Bar Harbor, ME) and maintained on a C57BL/6/J background. 129S2/SvHsd females were from Harlan Olac. The cross between *Hdh*Q150 and *Hdac4*HET mice, both on a C57BL/6 background, was performed at Novartis. All animals had unlimited access to food and water, were subject to a 12-h light/dark cycle, and housing conditions and environmental enrichment were as previously described [59]. The R6/2 colony was kept on breeding chow (Special Diet Services, Witham, UK).

Genomic DNA was isolated from an ear-punch. R6/2 and *Hdh*Q150 mice were genotyped by PCR, and the CAG repeat length was measured as previously described [25]. PCR conditions for genotyping *Hdac4* knock-out mice were for WT band: Fw: CTTGTTGAGAACAAACTCCTGCAGCT, Rw: AGCCCTACACTAGTGTGTGTTACACA; for *Hdac4* mutant band: Fw: AGCCCTACACTAGTGTGTGTTACACA, Neo Rw: CCATGGATCCTGAGACTGGGG.

Cycling conditions were 4 min at 95°C, 35× (30 s at 95°C; 45 s at 60°C; 2 min at 72°C), 10 min at 72°C using Taq polymerase and buffer from Promega. The *Hdh*Q20, *Hdh*Q80 mice [60] were genotyped as described [61] using the Hotstart polymerase (Thermoscientific).

Dissected tissues were snap frozen in liquid nitrogen and stored at −80°C until further analysis.

### Behavioural and Physiological Assessment

All behavioural tests were performed as previously described, and the data were analysed by repeated measures general linear model ANOVA with the Greenhouse Geisser post hoc test using SPSS software [59]. Pair-wise statistical comparisons were corrected for multiple comparisons using Bonferroni post hoc test in SPSS. Survival was assessed blind to genotype, and mice were euthanized when they reached end-stage disease. The data are presented as Kaplan–Meier cumulative survival functions and statistically analysed by the log-rank test.

### RNA Extraction, Taqman Real-Time PCR, and Affymetrix Gene Expression Arrays

Total RNA was extracted with the mini-RNA kit accordingly to manufacturer instructions (Qiagen). Reverse transcription (RT) was performed using MMLV superscript reverse transcriptase (Invitrogen) and random hexamers (Operon), and all Taqman-qPCR reactions were performed using the Chromo4 Real-Time PCR Detector (BioRad) as described [35]. Expression level of the gene-of-interest was normalised to the geometric mean of three endogenous housekeeping genes (Primer Design) as described [35]. The primer and probe sequences are detailed in Table S3.

For the Affymetrix arrays, biotinylated cRNAs were prepared from 200 ng total RNA using the GeneChip 3′ IVT Express Kit (Affymetrix) following the manufacturer's instructions. cRNA (15 µg) was hybridized to GeneChip Mouse Genome 430 version 2.0 Arrays (Affymetrix) and processed, stained, and scanned according to the manufacturer's recommendations. The quality of input RNAs and cRNAs was verified with the Bioanalyzer 2100 (Agilent Technologies) before use. Microarray quality control was performed using the software package provided on RACE [62]. Chips with a median normalized unscaled standard error greater than 1.05 were excluded. Affymetrix annotations (version 3.0) were used for probeset-to-gene assignments. Two-tailed *t* test was performed to assess the differences in gene expression between groups for each genotype (WT *n* = 8; R6/2 *n* = 9; *Hdac4*HET *n* = 8; Dble *n* = 9). Corrections for multiple testing were performed using the false discovery rate (FDR) according to Benjamini and Hochberg [63] with a significance threshold of *p*<0.05. The array datasets can be found at NCBI GEO accession number GSE38237.

### Antibodies, Western Blotting, and Seprion ELISA

All primary and secondary antibodies used in this study are presented in Table S4. Preparation of protein lysates and western blotting were as described previously [11]. In general, 20 µg protein lysate was fractionated on 10% SDS-PAGE gels. Aggregates were captured in Seprion ligand-coated plates (Microsens) and detected using the S830 sheep polyclonal or MW8 mouse monoclonal antibodies as described [25].

### TR-FRET

Sample preparation and the TR-FRET assay were performed as previously described [26].

### Nuclear Cytoplasmic Fractions and Agarose Gel Electrophoresis for Resolving Aggregates (AGERA)

Nuclear and cytoplasmic fractions were prepared as previously described [64], and their purity was determined by immunoblotting with antibodies to anti-histone H3 and α-tubulin. The AGERA assay was performed as described [65]. In general 100 µg of nuclear or cytoplasmic fractions, isolated from whole snap frozen brains, were loaded in nonreducing Laemmli buffer onto 1.5% agarose gels supplemented with 0.1% SDS and run at 3 V/cm followed by western blotting and immunodetection with anti-HTT (S830) antibodies. The high molecular weight protein marker was from Invitrogen.

### Co-Immunoprecipitation

Co-immunoprecipitation was performed as previously described [66]. Briefly, protein lysates were prepared from whole brains in HEPES buffer and incubated with protein-G Dynabeads (Invitrogen) overnight at 4°C on a rotating platform.

### Plasmids and GST Pull-Downs

The full-length mouse *Hdac4* gene in pCMV6 was obtained from Origene. This was amplified by PCR using high fidelity polymerase (Roche) and subsequently cloned into pCR2-Topo (Invitrogen) accordingly to the manufacturer's instructions. The detailed protocol used for GST pull-downs is available in the Text S1.

### Immunohistochemistry and Confocal Microscopy

For immunohistochemical studies, brains were frozen in isopentane at −50°C and stored at −80°C until further analysis. We cut 10–15 µm sections using a cryostat (Bright instruments), air dried and immersed them in 4% PFA in PBS for 15 min, and washed them for 3×5 min in 0.1% PBS-Triton X-100. Blocking was achieved by incubation with 5% BSA-C (Aurion) in 0.1% PBS-Triton X-100 for at least 30 min at RT. Immunolabelling with primary antibodies in 0.1% PBS-Triton X-100, 1% BSA-C (HDAC4, S830) was completed by overnight incubation in a humidity box at 4°C. Sections were washed 3× in PBS, incubated for 60 min at RT in a dark box with the appropriate combinations of secondary antibodies diluted in PBS, washed 3× in PBS, and counterstained with DAPI (Invitrogen). Sections were mounted in Vectashield mounting medium (Vector Laboratories). Sections were examined using the Leica TCS SP4 laser scanning confocal microscope and analysed with Leica Application Suite (LAS) v5 (Leica Microsystems, Heidelberg, Germany).

### Electrophysiological Recordings

Detailed procedures for acute striatal slice preparation, patch-clamp recordings, and the isolation of miniature excitatory postsynaptic currents (mEPSCs), along with the appropriate statistical analysis, can be found in Text S1.

### Statistical Analysis

All data were analysed with Microsoft Office Excel and two-tailed Student's *t* test or as otherwise stated.

## Supporting Information

Figure S1 **HDAC4 reduction does not alter the expression levels of** ***HTT*** **exon 1, endogenous** ***Htt*** **, or other** ***Hdacs*** **.** (A) Taqman qPCR showed no difference in *HTT* exon 1 transgene levels in the cerebellum or in the striatum of R6/2 and Dble::R6/2. (B) There was no difference in endogenous *Htt* levels in the cerebellum or in the striatum of WT, *Hdac*4HET, R6/2, and Dble::R6/2 mice as determined by Taqman qPCR. (C) Taqman qPCR showed that the cerebellar transcript level of *Hdac4* was decreased in *Hdac4*HET and Dble::R6/2 mice, but that the expression level of other *Hdacs* did not differ from WT in the cerebellum of *Hdac*4HET, R6/2, and Dble::R6/2. (D) Taqman qPCR showed that the striatal transcript level of *Hdac4* was decreased in *Hdac4*HET and Dble mice, but that the expression level of other *Hdacs* did not differ from WT in the striatum of *Hdac*4HET, R6/2, and Dble::R6/2. Taqman qPCR values were normalized to the geometric mean of three housekeeping genes: *Atp5b*, *Canx*, and *Eif4a* (for cerebellum) and *Atp5b*, *Yhwaz*, and *Ubc* (for striatum). Error bars are SEM. *p* values were calculated using Student's *t* test (*n* = 8). **p<0.01, ***p<0.001.
(TIF)

Figure S2 **HDAC4 knock-down delays aggregate formation in multiple CNS tissues.** (A) Seprion ligand ELISA was used to quantify the aggregate load in the brain stem, hippocampus, cerebellum, and striatum of R6/2 and Dble::R6/2 mice at 4, 9, and 15 wk of age. R6/2::Dble values were plotted as a percentage of R6/2 aggregate load (*n* = 6). (B) TR-FRET was used to determine the levels of soluble exon 1 HTT in the brain stem, hippocampus, and cerebellum of R6/2 and Dble mice at 4, 9, and 15 wk of age (*n* = 6). (C) Western blot demonstrating that the H92 antibody detects HDAC4 as the signal is absent from HDAC4 knock-out tissue. Immunoprecipitation with the HDAC4 antibodies H92, DM-15, and CS2072 demonstrates that they are all capable of immunoprecipitating HDAC4 as detected with H92. ID, immunodetection. (D) Western blot of detergent-insoluble high molecular weight aggregates isolated from the nuclear and cytoplasmic fractions of R6/2 and Dble::R6/2 (Dble) brains at 4 wk of age, resolved by agarose gel electrophoresis (AGERA), and immunodetected with the S830 antibody (*n* = 8). The purity of the fractions was demonstrated by western blotting with α-tubulin and histone H3. (E) Western blot of HDAC4 protein levels in the cytoplasmic fractions of R6/2 and Dble::R6/2 (Dble) brains at 4 wk of age. HDAC4 levels in the cytoplasmic fractions were measured by densitometry and calculated relative to α-tubulin. (F) Seprion ligand ELISA was used to quantify aggregate load in the cortex of R6/2-129S mice as compared to R6/2-CBF mice at 9 wk of age. R6/2-129S values were plotted as a percentage of aggregate load in R6/2-CBF mice (*n* = 7). R6/2-129S mice have the same *Hdac4* haplotype as Dble mice but with a functional *Hdac4* gene. Error bars are SEM. *p* values were calculated using Student's *t* test.
(TIF)

Figure S3 **HDAC4 associates with exon 1 HTT and co-localizes with cytoplasmic inclusions and with synaptic markers.** (A) Validation of the specificity of the HDAC4 antibodies used for immunohistochemistry. Representative immunofluorescent images of cortex from WT (A) and *Hdac4*KO (B) mice stained for HDAC4 at P3 and counterstained with DAPI. Scale bar, 10 µm. (B) Representative immunofluorescent images of the striatum from 14-wk-old WT and R6/2 mice immunostained for mutant HTT (S830) and HDAC4 (CS2072), and counterstained with DAPI. Scale bar, 15 µm. (C) Representative confocal images of the cortex from 14-wk WT mice. Sections were stained for synaptophysin and HDAC4 or for PSD95 and HDAC4 and counterstained with DAPI. There was a considerable degree of co-localisation between HDAC4 and the synaptic markers. Scale bar, 10 µm.
(TIF)

Table S1 **Summary of the number of mice per genotype used in all studies and their CAG repeat sizes. SD, standard deviation.**
(DOCX)

Table S2 **Summary of neurological and physiological phenotypes as assessed by the SHIRPA protocol.**
(DOCX)

Table S3 **Summary of the Taq-man assays used in this study.**
(DOCX)

Table S4 **Summary of the antibodies used in this study.** WB, western blotting; IP, immunoprecipitation; IHC, immunohistochemistry; SEPRION, seprion ligand ELISA for aggregated huntingtin; TR-FRET, time resolved Förster resonance energy transfer for soluble huntingtin.
(DOCX)

Text S1 **Supporting materials and methods.** Plasmids and GST pull-downs and electrophysiological recordings.
(DOCX)

Video S1 **Comparison of the gait of an R6/2 mouse (agouti) and his Dble::R6/2 littermate (black) at 14 wk of age (males).**
(MP4)

Video S2 **Comparison of the appearance of an R6/2 mouse (agouti) and his Dble::R6/2 littermate (black) at 14 wk of age (males).** Note improvements in body tone, hunched back, piloerection, and general appearance.
(M4V)

## Abbreviations

AGERA: agarose gel electrophoresis resolving aggregates
BDNF: brain derived neurotrophic factor
EPSCs: excitatory post-synaptic currents
GST: glutathione-S-transferase
HD: Huntington's disease
HDAC: histone deacetylase
*Hdac4*HET: *Hdac4* knock-out heterozygote
*Hdac4*KO: *Hdac4* knock-out
HTT: huntingtin
MEF: myocyte enhancing factor
mEPSC: miniature EPSC
MSN: medium spiny neuron
polyQ: polyglutamine
REST: RE1 silencing transcription factor
RMP: resting membrane potential
SAHA: suberoylanilide hydroxamic acid
SNP: single nucleotide polymorphism
TR-FRET: time resolved Förster resonance energy transfer

## References

[1] Novak MJ, Tabrizi SJ (2010) Huntington's disease. BMJ 340: c3109. 20591965 10.1136/bmj.c3109

[2] Landles C, Bates GP (2004) Huntingtin and the molecular pathogenesis of Huntington's disease. Fourth in molecular medicine review series. EMBO Rep 5: 958–963. 15459747 10.1038/sj.embor.7400250 PMC1299150

[3] Cha JH (2007) Transcriptional signatures in Huntington's disease. Prog Neurobiol 83: 228–248. 17467140 10.1016/j.pneurobio.2007.03.004 PMC2449822

[4] Butler R, Bates GP (2006) Histone deacetylase inhibitors as therapeutics for polyglutamine disorders. Nat Rev Neurosci 7: 784–796. 16988654 10.1038/nrn1989

[5] Kazantsev AG, Thompson LM (2008) Therapeutic application of histone deacetylase inhibitors for central nervous system disorders. Nat Rev Drug Discovery 7: 854–868. 18827828 10.1038/nrd2681

[6] Haberland M, Montgomery RL, Olson EN (2009) The many roles of histone deacetylases in development and physiology: implications for disease and therapy. Nat Rev Genet 10: 32–42. 19065135 10.1038/nrg2485 PMC3215088

[7] Hockly E, Richon VM, Woodman B, Smith DL, Zhou X, et al (2003) Suberoylanilide hydroxamic acid, a histone deacetylase inhibitor, ameliorates motor deficits in a mouse model of Huntington's disease. Proc Natl Acad Sci U S A 100: 2041–2046. 12576549 10.1073/pnas.0437870100 PMC149955

[8] Marks PA, Xu WS (2009) Histone deacetylase inhibitors: potential in cancer therapy. J Cell Biochem 107: 600–608. 19459166 10.1002/jcb.22185 PMC2766855

[9] Scognamiglio A, Nebbioso A, Manzo F, Valente S, Mai A, et al (2008) HDAC-class II specific inhibition involves HDAC proteasome-dependent degradation mediated by RANBP2. Biochim Biophys Acta 1783: 2030–2038. 18691615 10.1016/j.bbamcr.2008.07.007

[10] Benn CL, Butler R, Mariner L, Nixon J, Moffitt H, et al (2009) Genetic knock-down of HDAC7 does not ameliorate disease pathogenesis in the R6/2 mouse model of Huntington's disease. PLoS ONE 4 (6) e5747 doi:10.1371/journal.pone.0005747 19484127 10.1371/journal.pone.0005747 PMC2684627

[11] Mielcarek M, Benn CL, Franklin SA, Smith DL, Woodman B, et al (2011) SAHA decreases HDAC 2 and 4 levels in vivo and improves molecular phenotypes in the R6/2 mouse model of Huntington's disease. Plos ONE 6 (11) e27746 doi:10.1371/journal.pone.0027746 22140466 10.1371/journal.pone.0027746 PMC3225376

[12] Verdin E, Dequiedt F, Kasler HG (2003) Class II histone deacetylases: versatile regulators. Trends Genet 19: 286–293. 12711221 10.1016/S0168-9525(03)00073-8

[13] Bolger TA, Yao TP (2005) Intracellular trafficking of histone deacetylase 4 regulates neuronal cell death. J Neurosci 25: 9544–9553. 16221865 10.1523/JNEUROSCI.1826-05.2005 PMC6725694

[14] Mao Z, Bonni A, Xia F, Nadal-Vicens M, Greenberg ME (1999) Neuronal activity-dependent cell survival mediated by transcription factor MEF2. Science 286: 785–790. 10531066 10.1126/science.286.5440.785

[15] Guo L, Han A, Bates DL, Cao J, Chen L (2007) Crystal structure of a conserved N-terminal domain of histone deacetylase 4 reveals functional insights into glutamine-rich domains. Proc Natl Acad Sci U S A 104: 4297–4302. 17360518 10.1073/pnas.0608041104 PMC1838596

[16] Fiumara F, Fioriti L, Kandel ER, Hendrickson WA (2010) Essential role of coiled coils for aggregation and activity of Q/N-rich prions and PolyQ proteins. Cell 143: 1121–1135. 21183075 10.1016/j.cell.2010.11.042 PMC3472970

[17] Mangiarini L, Sathasivam K, Seller M, Cozens B, Harper A, et al (1996) Exon 1 of the HD gene with an expanded CAG repeat is sufficient to cause a progressive neurological phenotype in transgenic mice. Cell 87: 493–506. 8898202 10.1016/s0092-8674(00)81369-0

[18] Lin CH, Tallaksen-Greene S, Chien WM, Cearley JA, Jackson WS, et al (2001) Neurological abnormalities in a knock-in mouse model of Huntington's disease. Hum Mol Genet 10: 137–144. 11152661 10.1093/hmg/10.2.137

[19] Woodman B, Butler R, Landles C, Lupton MK, Tse J, et al (2007) The Hdh(Q150/Q150) knock-in mouse model of HD and the R6/2 exon 1 model develop comparable and widespread molecular phenotypes. Brain Res Bull 72: 83–97. 17352931 10.1016/j.brainresbull.2006.11.004

[20] Sathasivam K, Neueder A, Gipson TA, Landles C, Benjamin AC, et al (2013) Aberrant splicing of HTT generates the pathogenic exon 1 protein in Huntington disease. Proc Natl Acad Sci U S A 110: 2366–2370. 23341618 10.1073/pnas.1221891110 PMC3568346

[21] Vega RB, Matsuda K, Oh J, Barbosa AC, Yang XL, et al (2004) Histone deacetylase 4 controls chondrocyte hypertrophy during skeletogenesis. Cell 119: 555–566. 15537544 10.1016/j.cell.2004.10.024

[22] Martin M, Kettmann R, Dequiedt F (2007) Class IIa histone deacetylases: regulating the regulators. Oncogene 26: 5450–5467. 17694086 10.1038/sj.onc.1210613

[23] Haberland M, Arnold MA, McAnally J, Phan D, Kim Y, et al (2007) Regulation of HDAC9 gene expression by MEF2 establishes a negative-feedback loop in the transcriptional circuitry of muscle differentiation. Mol Cell Biol 27: 518–525. 17101791 10.1128/MCB.01415-06 PMC1800816

[24] Mihaylova MM, Vasquez DS, Ravnskjaer K, Denechaud PD, Yu RT, et al (2011) Class IIa histone deacetylases are hormone-activated regulators of FOXO and mammalian glucose homeostasis. Cell 145: 607–621. 21565617 10.1016/j.cell.2011.03.043 PMC3117637

[25] Sathasivam K, Lane A, Legleiter J, Warley A, Woodman B, et al (2010) Identical oligomeric and fibrillar structures captured from the brains of R6/2 and knock-in mouse models of Huntington's disease. Hum Mol Genet 19: 65–78. 19825844 10.1093/hmg/ddp467 PMC2792149

[26] Weiss A, Abramowski D, Bibel M, Bodner R, Chopra V, et al (2009) Single-step detection of mutant huntingtin in animal and human tissues: a bioassay for Huntington's disease. Anal Biochem 395: 8–15. 19664996 10.1016/j.ab.2009.08.001

[27] Moumne L, Campbell K, Howland D, Ouyang Y, Bates GP (2012) Genetic knock-down of HDAC3 does not modify disease-related phenotypes in a mouse model of Huntington's disease. PLoS ONE 7 (2) e31080 doi:10.1371/journal.pone.0031080 22347433 10.1371/journal.pone.0031080 PMC3275566

[28] Bobrowska A, Donmez G, Weiss A, Guarente L, Bates G (2012) SIRT2 ablation has no effect on tubulin acetylation in brain, cholesterol biosynthesis or the progression of Huntington's disease phenotypes in vivo. PLoS ONE 7 (4) e34805 doi:10.1371/journal.pone.0034805 22511966 10.1371/journal.pone.0034805 PMC3325254

[29] Bobrowska A, Paganetti P, Matthias P, Bates GP (2011) Hdac6 knock-out increases tubulin acetylation but does not modify disease progression in the R6/2 mouse model of Huntington's disease. PLoS ONE 6 (6) e20696 doi:10.1371/journal.pone.0020696 21677773 10.1371/journal.pone.0020696 PMC3108987

[30] Zourlidou A, Gidalevitz T, Kristiansen M, Landles C, Woodman B, et al (2007) Hsp27 overexpression in the R6/2 mouse model of Huntington's disease: chronic neurodegeneration does not induce Hsp27 activation. Hum Mol Genet 16: 1078–1090. 17360721 10.1093/hmg/ddm057

[31] Hay DG, Sathasivam K, Tobaben S, Stahl B, Marber M, et al (2004) Progressive decrease in chaperone protein levels in a mouse model of Huntington's disease and induction of stress proteins as a therapeutic approach. Hum Mol Genet 13: 1389–1405. 15115766 10.1093/hmg/ddh144

[32] Smith DL, Woodman B, Mahal A, Sathasivam K, Ghazi-Noori S, et al (2003) Minocycline and doxycycline are not beneficial in a model of Huntington's disease. Ann Neurol 54: 186–196. 12891671 10.1002/ana.10614

[33] Hockly E, Tse J, Barker AL, Moolman DL, Beunard JL, et al (2006) Evaluation of the benzothiazole aggregation inhibitors riluzole and PGL-135 as therapeutics for Huntington's disease. Neurobiol Dis 21: 228–236. 16111888 10.1016/j.nbd.2005.07.007

[34] Kuhn A, Goldstein DR, Hodges A, Strand AD, Sengstag T, et al (2007) Mutant huntingtin's effects on striatal gene expression in mice recapitulate changes observed in human Huntington's disease brain and do not differ with mutant huntingtin length or wild-type huntingtin dosage. Hum Mol Genet 16: 1845–1861. 17519223 10.1093/hmg/ddm133

[35] Benn CL, Fox H, Bates GP (2008) Optimisation of region-specific reference gene selection and relative gene expression analysis methods for pre-clinical trials of Huntington's disease. Mol Neurodegener 3: 17. 18954449 10.1186/1750-1326-3-17 PMC2584034

[36] Klapstein GJ, Fisher RS, Zanjani H, Cepeda C, Jokel ES, et al (2001) Electrophysiological and morphological changes in striatal spiny neurons in R6/2 Huntington's disease transgenic mice. J Neurophys 86: 2667–2677. 10.1152/jn.2001.86.6.2667 11731527

[37] Cepeda C, Hurst RS, Calvert CR, Hernandez-Echeagaray E, Nguyen OK, et al (2003) Transient and progressive electrophysiological alterations in the corticostriatal pathway in a mouse model of Huntington's disease. J Neurosci 23: 961–969. 12574425 10.1523/JNEUROSCI.23-03-00961.2003 PMC6741903

[38] Rogers DC, Fisher EM, Brown SD, Peters J, Hunter AJ, et al (1997) Behavioral and functional analysis of mouse phenotype: SHIRPA, a proposed protocol for comprehensive phenotype assessment. Mamm Genome 8: 711–713. 9321461 10.1007/s003359900551

[39] Zhao X, Ito A, Kane CD, Liao TS, Bolger TA, et al (2001) The modular nature of histone deacetylase HDAC4 confers phosphorylation-dependent intracellular trafficking. J Biol Chem 276: 35042–35048. 11470791 10.1074/jbc.M105086200

[40] Miska EA, Langley E, Wolf D, Karlsson C, Pines J, et al (2001) Differential localization of HDAC4 orchestrates muscle differentiation. Nucleic Acids Res 29: 3439–3447. 11504882 10.1093/nar/29.16.3439 PMC55849

[41] Darcy MJ, Calvin K, Cavnar K, Ouimet CC (2010) Regional and subcellular distribution of HDAC4 in mouse brain. J Comp Neurol 518: 722–740. 20034059 10.1002/cne.22241

[42] Mielcarek M, Seredenina T, Stokes M, Osborne GF, Landles C, et al (2013) HDAC4 does not act as a protein deacetylase in the postnatal brain *in vivo* . PLoS ONE in press. 10.1371/journal.pone.0080849 PMC3838388 24278330

[43] Bottomley MJ, Lo Surdo P, Di Giovine P, Cirillo A, Scarpelli R, et al (2008) Structural and functional analysis of the human HDAC4 catalytic domain reveals a regulatory structural zinc-binding domain. J Biol Chem 283: 26694–26704. 18614528 10.1074/jbc.M803514200 PMC3258910

[44] Zuccato C, Tartari M, Crotti A, Goffredo D, Valenza M, et al (2003) Huntingtin interacts with REST/NRSF to modulate the transcription of NRSE-controlled neuronal genes. Nat Genet 35: 76–83. 12881722 10.1038/ng1219

[45] Li H, Li SH, Cheng AL, Mangiarini L, Bates GP, et al (1999) Ultrastructural localization and progressive formation of neuropil aggregates in Huntington's disease transgenic mice. Hum Mol Genet 8: 1227–1236. 10369868 10.1093/hmg/8.7.1227

[46] Benn CL, Landles C, Li H, Strand AD, Woodman B, et al (2005) Contribution of nuclear and extranuclear polyQ to neurological phenotypes in mouse models of Huntington's disease. Hum Mol Genet 14: 3065–3078. 16183657 10.1093/hmg/ddi340

[47] Gutekunst CA, Li SH, Yi H, Mulroy JS, Kuemmerle S, et al (1999) Nuclear and neuropil aggregates in Huntington's disease: relationship to neuropathology. J Neurosci 19: 2522–2534. 10087066 10.1523/JNEUROSCI.19-07-02522.1999 PMC6786077

[48] Kikis EA, Gidalevitz T, Morimoto RI (2010) Protein homeostasis in models of aging and age-related conformational disease. Adv Exp Med Biol 694: 138–159. 20886762 10.1007/978-1-4419-7002-2_11 PMC3402352

[49] Labbadia J, Cunliffe H, Weiss A, Katsyuba E, Sathasivam K, et al (2011) Altered chromatin architecture underlies progressive impairment of the heat shock response in mouse models of Huntington disease. J Clin Invest 121: 3306–3319. 21785217 10.1172/JCI57413 PMC3148745

[50] Labbadia J, Novoselov SS, Bett JS, Weiss A, Paganetti P, et al (2012) Suppression of protein aggregation by chaperone modification of high molecular weight complexes. Brain 135: 1180–1196. 22396390 10.1093/brain/aws022 PMC3326252

[51] Gunawardena S, Goldstein LS (2005) Polyglutamine diseases and transport problems: deadly traffic jams on neuronal highways. Arch Neurol 62: 46–51. 15642849 10.1001/archneur.62.1.46

[52] Sassone J, Colciago C, Cislaghi G, Silani V, Ciammola A (2009) Huntington's disease: the current state of research with peripheral tissues. Exp Neurol 219: 385–397. 19460373 10.1016/j.expneurol.2009.05.012

[53] Moresi V, Williams AH, Meadows E, Flynn JM, Potthoff MJ, et al (2010) Myogenin and class II HDACs control neurogenic muscle atrophy by inducing E3 ubiquitin ligases. Cell 143: 35–45. 20887891 10.1016/j.cell.2010.09.004 PMC2982779

[54] Sah DW, Aronin N (2011) Oligonucleotide therapeutic approaches for Huntington disease. J Clin Invest 121: 500–507. 21285523 10.1172/JCI45130 PMC3026739

[55] Kordasiewicz HB, Stanek LM, Wancewicz EV, Mazur C, McAlonis MM, et al (2012) Sustained therapeutic reversal of Huntington's disease by transient repression of huntingtin synthesis. Neuron 74: 1031–1044. 22726834 10.1016/j.neuron.2012.05.009 PMC3383626

[56] Yu D, Pendergraff H, Liu J, Kordasiewicz HB, Cleveland DW, et al (2012) Single-stranded RNAs use RNAi to potently and allele-selectively inhibit mutant huntingtin expression. Cell 150: 895–908. 22939619 10.1016/j.cell.2012.08.002 PMC3444165

[57] Takahashi-Fujigasaki J, Fujigasaki H (2006) Histone deacetylase (HDAC) 4 involvement in both Lewy and Marinesco bodies. Neuropath Appl Neurobiol 32: 562–566. 10.1111/j.1365-2990.2006.00733.x 16972890

[58] Kilgore M, Miller CA, Fass DM, Hennig KM, Haggarty SJ, et al (2010) Inhibitors of class 1 histone deacetylases reverse contextual memory deficits in a mouse model of Alzheimer's disease. Neuropsychopharmacol 35: 870–880. 10.1038/npp.2009.197 PMC3055373 20010553

[59] Hockly E, Woodman B, Mahal A, Lewis CM, Bates G (2003) Standardization and statistical approaches to therapeutic trials in the R6/2 mouse. Brain Res Bull 61: 469–479. 13679245 10.1016/s0361-9230(03)00185-0

[60] Wheeler VC, White JK, Gutekunst CA, Vrbanac V, Weaver M, et al (2000) Long glutamine tracts cause nuclear localization of a novel form of huntingtin in medium spiny striatal neurons in HdhQ92 and HdhQ111 knock- in mice. Hum Mol Genet 9: 503–513. 10699173 10.1093/hmg/9.4.503

[61] White JK, Auerbach W, Duyao MP, Vonsattel JP, Gusella JF, et al (1997) Huntingtin is required for neurogenesis and is not impaired by the Huntington's disease CAG expansion. Nat Genet 17: 404–410. 9398841 10.1038/ng1297-404

[62] Psarros M, Heber S, Sick M, Thoppae G, Harshman K, et al (2005) RACE: Remote Analysis Computation for gene Expression data. Nucleic Acids Res 33: W638–643. 15980552 10.1093/nar/gki490 PMC1160250

[63] Hochberg Y, Benjamini Y (1990) More powerful procedures for multiple significance testing. Stat Med 9: 811–818. 2218183 10.1002/sim.4780090710

[64] Davies SW, Sathasivam K, Hobbs C, Doherty P, Mangiarini L, et al (1999) Detection of polyglutamine aggregation in mouse models. Methods Enzymol 309: 687–701. 10507055 10.1016/s0076-6879(99)09045-x

[65] Weiss A, Klein C, Woodman B, Sathasivam K, Bibel M, et al (2008) Sensitive biochemical aggregate detection reveals aggregation onset before symptom development in cellular and murine models of Huntington's disease. J Neurochem 104: 846–858. 17986219 10.1111/j.1471-4159.2007.05032.x

[66] Landles C, Sathasivam K, Weiss A, Woodman B, Moffitt H, et al (2010) Proteolysis of mutant huntingtin produces an exon 1 fragment that accumulates as an aggregated protein in neuronal nuclei in Huntington disease. J Biol Chem 285: 8808–8823. 20086007 10.1074/jbc.M109.075028 PMC2838303

