# Supplementary figures and images for: HDAC4 Reduction: A Novel Therapeutic Strategy to Target Cytoplasmic Huntingtin and Ameliorate Neurodegeneration
Source: PLoS Biol. 2013 Nov 26;11(11):e1001717. doi: 10.1371/journal.pbio.1001717 (PMC3841096; doi:10.1371/journal.pbio.1001717)

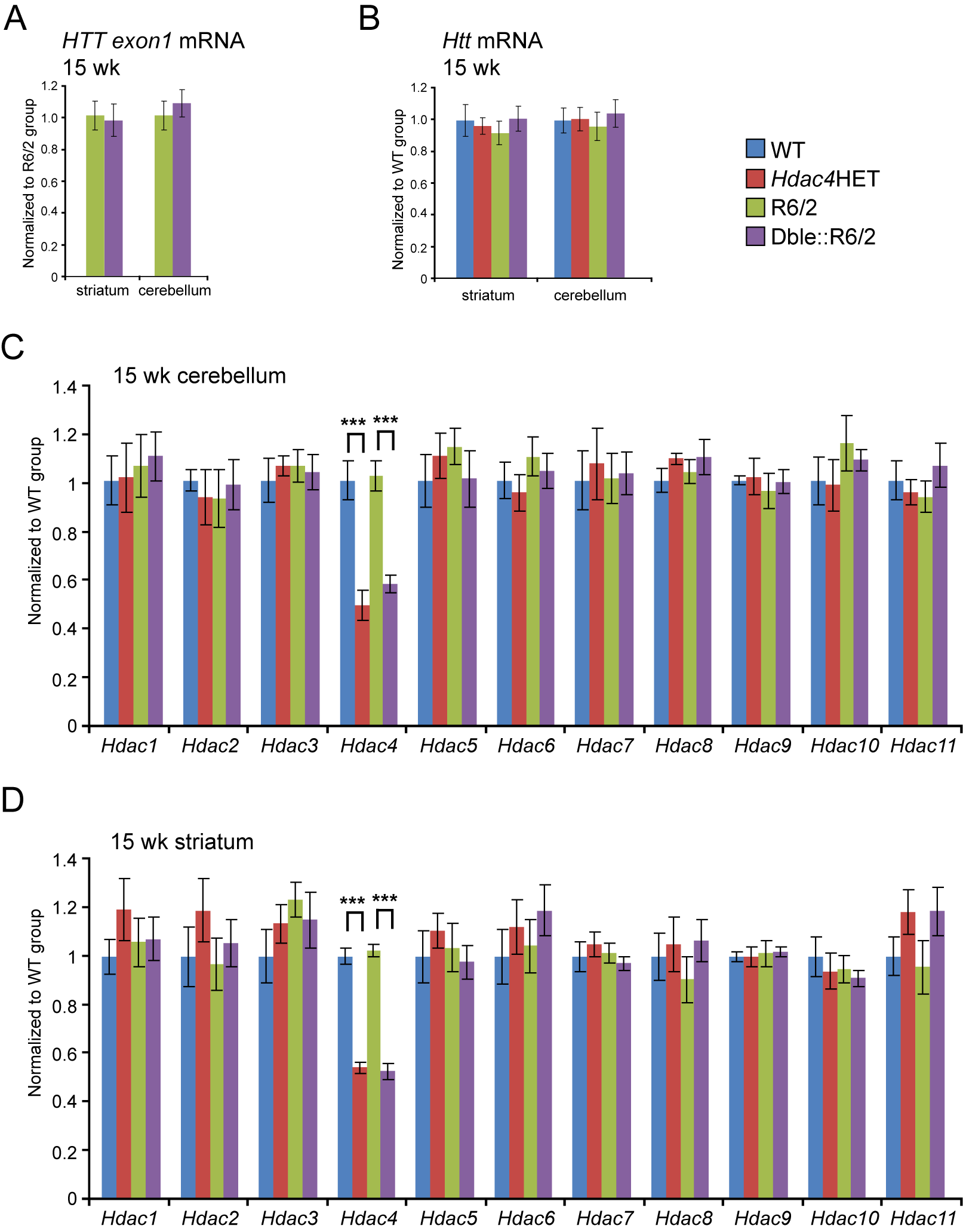

Supplement: Figure S1 — HDAC4 reduction does not alter the expression levels of HTT exon 1, endogenous Htt , or other Hdacs . (A) Taqman qPCR showed no difference in HTT exon 1 transgene levels in the cerebellum or in the striatum of R6/2 and Dble::R6/2. (B) There was no difference in endogenous Htt levels in the cerebellum or in the striatum of WT, Hdac4HET, R6/2, and Dble::R6/2 mice as determined by Taqman qPCR. (C) Taqman qPCR showed that the cerebellar transcript level of Hdac4 was decreased in Hdac4HET and Dble::R6/2 mice, but that the expression level of other Hdacs did not differ from WT in the cerebellum of Hdac4HET, R6/2, and Dble::R6/2. (D) Taqman qPCR showed that the striatal transcript level of Hdac4 was decreased in Hdac4HET and Dble mice, but that the expression level of other Hdacs did not differ from WT in the striatum of Hdac4HET, R6/2, and Dble::R6/2. Taqman qPCR values were normalized to the geometric mean of three housekeeping genes: Atp5b, Canx, and Eif4a (for cerebellum) and Atp5b, Yhwaz, and Ubc (for striatum). Error bars are SEM. p values were calculated using Student's t test (n = 8). **p<0.01, ***p<0.001. (TIF) [file pbio.1001717.s001.tif]

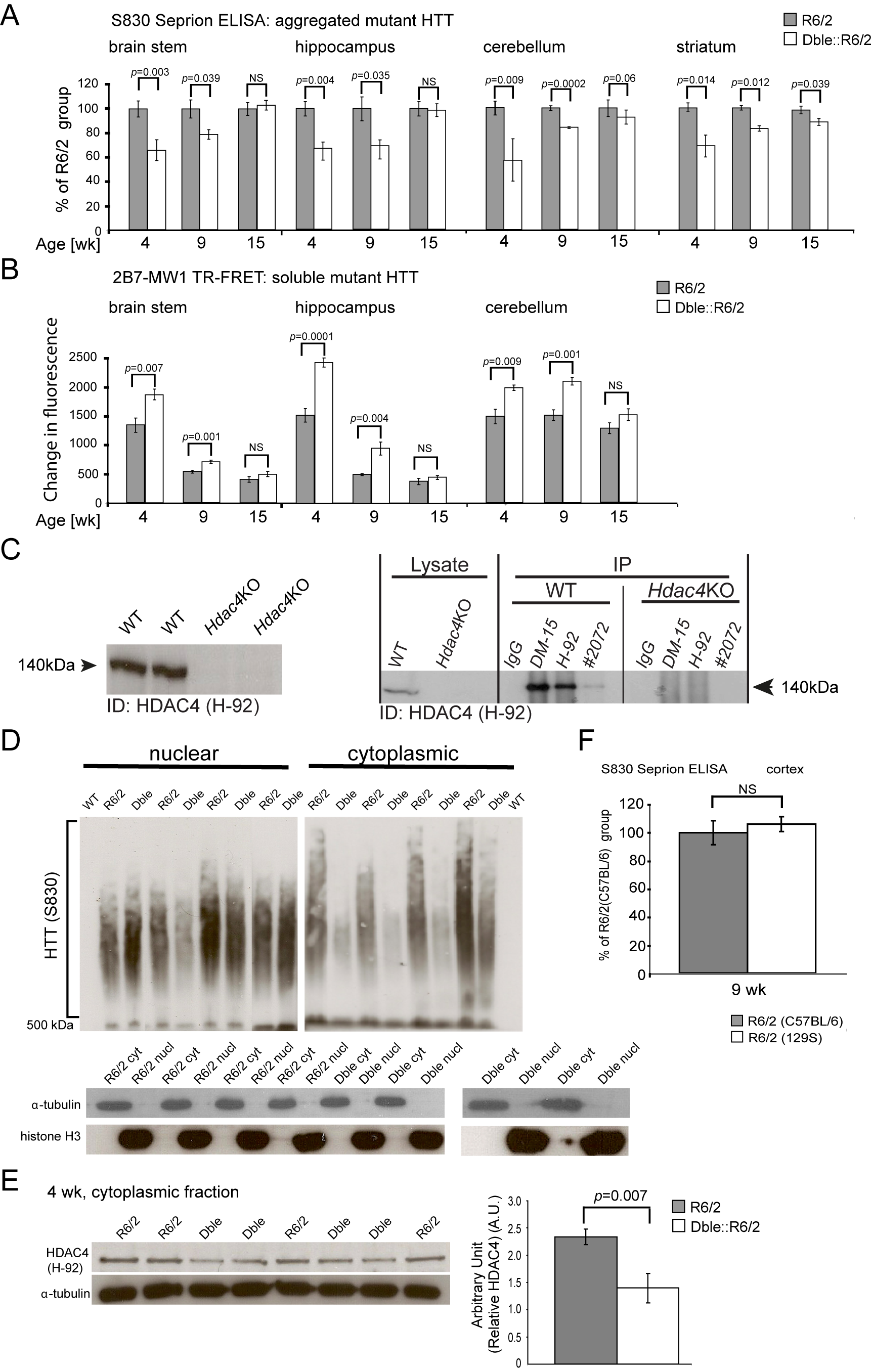

Supplement: Figure S2 — HDAC4 knock-down delays aggregate formation in multiple CNS tissues. (A) Seprion ligand ELISA was used to quantify the aggregate load in the brain stem, hippocampus, cerebellum, and striatum of R6/2 and Dble::R6/2 mice at 4, 9, and 15 wk of age. R6/2::Dble values were plotted as a percentage of R6/2 aggregate load (n = 6). (B) TR-FRET was used to determine the levels of soluble exon 1 HTT in the brain stem, hippocampus, and cerebellum of R6/2 and Dble mice at 4, 9, and 15 wk of age (n = 6). (C) Western blot demonstrating that the H92 antibody detects HDAC4 as the signal is absent from HDAC4 knock-out tissue. Immunoprecipitation with the HDAC4 antibodies H92, DM-15, and CS2072 demonstrates that they are all capable of immunoprecipitating HDAC4 as detected with H92. ID, immunodetection. (D) Western blot of detergent-insoluble high molecular weight aggregates isolated from the nuclear and cytoplasmic fractions of R6/2 and Dble::R6/2 (Dble) brains at 4 wk of age, resolved by agarose gel electrophoresis (AGERA), and immunodetected with the S830 antibody (n = 8). The purity of the fractions was demonstrated by western blotting with α-tubulin and histone H3. (E) Western blot of HDAC4 protein levels in the cytoplasmic fractions of R6/2 and Dble::R6/2 (Dble) brains at 4 wk of age. HDAC4 levels in the cytoplasmic fractions were measured by densitometry and calculated relative to α-tubulin. (F) Seprion ligand ELISA was used to quantify aggregate load in the cortex of R6/2-129S mice as compared to R6/2-CBF mice at 9 wk of age. R6/2-129S values were plotted as a percentage of aggregate load in R6/2-CBF mice (n = 7). R6/2-129S mice have the same Hdac4 haplotype as Dble mice but with a functional Hdac4 gene. Error bars are SEM. p values were calculated using Student's t test. (TIF) [file pbio.1001717.s002.tif]

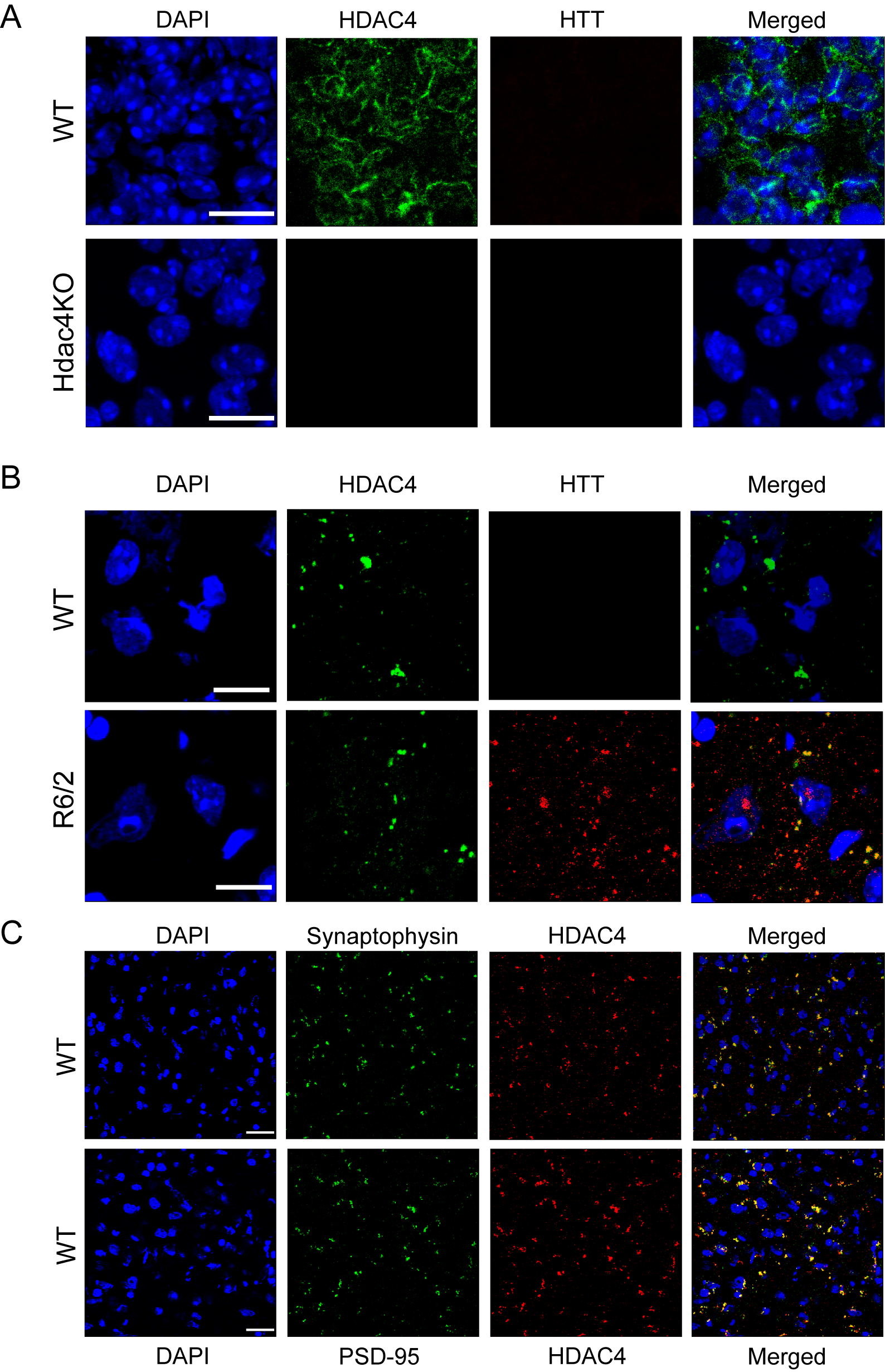

Supplement: Figure S3 — HDAC4 associates with exon 1 HTT and co-localizes with cytoplasmic inclusions and with synaptic markers. (A) Validation of the specificity of the HDAC4 antibodies used for immunohistochemistry. Representative immunofluorescent images of cortex from WT (A) and Hdac4KO (B) mice stained for HDAC4 at P3 and counterstained with DAPI. Scale bar, 10 µm. (B) Representative immunofluorescent images of the striatum from 14-wk-old WT and R6/2 mice immunostained for mutant HTT (S830) and HDAC4 (CS2072), and counterstained with DAPI. Scale bar, 15 µm. (C) Representative confocal images of the cortex from 14-wk WT mice. Sections were stained for synaptophysin and HDAC4 or for PSD95 and HDAC4 and counterstained with DAPI. There was a considerable degree of co-localisation between HDAC4 and the synaptic markers. Scale bar, 10 µm. (TIF) [file pbio.1001717.s003.tif]
