# Supplementary material for: HDAC4 Reduction: A Novel Therapeutic Strategy to Target Cytoplasmic Huntingtin and Ameliorate Neurodegeneration
Source: PLoS Biol. 2013 Nov 26;11(11):e1001717. doi: 10.1371/journal.pbio.1001717 (PMC3841096; doi:10.1371/journal.pbio.1001717)
Supplement: Table S1 — Summary of the number of mice per genotype used in all studies and their CAG repeat sizes. SD, standard deviation. (DOCX) [file pbio.1001717.s004.docx]

| Study | Genotype | Total # mice | Mean CAG repeat Size | ±SD |
| --- | --- | --- | --- | --- |
| Behavioural tests: rotarod, body weight | WT | 31 | - | - |
|  | *Hdac4*HET | 22 | - | - |
|  | R6/2 | 21 | 202 | 2.1 |
|  | Dble::R62 | 20 | 202 | 2.7 |
| Survival test | WT | 18 | - | - |
|  | *Hdac4*HET | 22 | - | - |
|  | R6/2 | 18 | 202 | 2.2 |
|  | Dble::R6/2 | 14 | 202 | 2.6 |
| Electrophysiology 7-8 weeks | WT | 9 | - | - |
|  | *Hdac4*HET | 6 | - | - |
|  | R6/2 | 14 | 200 | 2.1 |
|  | Dble::R6/2 | 18 | 200 | 2.7 |
| Electrophysiology 10-12 weeks | WT | 10 | - | - |
|  | *Hdac4*HET | 12 | - | - |
|  | R6/2 | 11 | 200 | 2.1 |
|  | Dble::R6/2 | 9 | 200 | 1.7 |
| Molecular biology tests  R6/2 x :*Hdac4*HET  Various studies: 4-14 weeks | WT | 44 | - | - |
|  | *Hdac4*HET | 44 | - | - |
|  | R6/2 | 72 | 203 | 5.6 |
|  | Dble::R6/2 | 72 | 203 | 6.1 |
| Molecular biology tests  *Hdh*Q150 x :*Hdac4*HET  6 and 10 months | WT | 17 | - | - |
|  | *Hdac4*HET | 16 | - | - |
|  | *Hdh* Q150 | 18 | 160 | 13.7 |
|  | Dble::*Hdh*Q150 | 20 | 158 | 15 |
