# Supplementary material for: HDAC4 Reduction: A Novel Therapeutic Strategy to Target Cytoplasmic Huntingtin and Ameliorate Neurodegeneration
Source: PLoS Biol. 2013 Nov 26;11(11):e1001717. doi: 10.1371/journal.pbio.1001717 (PMC3841096; doi:10.1371/journal.pbio.1001717)
Supplement: Table S2 — Summary of neurological and physiological phenotypes as assessed by the SHIRPA protocol. (DOCX) [file pbio.1001717.s005.docx]

| Phenotype | Scoring system |
| --- | --- |
| **Locomotor Activity/sq in 30s** | Number of squares entered with all four feet in 30s |
| **Piloerection** | 0 - None, 1 - Present |
| **Tremor** | 0 - None, 1 - Present |
| **Hunched back** | 0 - None, 1 - Present |
| **Gait** | 0 - Fluid movement and approx. 3mm pelvic elevation; 1 - Lack of fluidity in movement |
| **Touch Escape** | 0 - No response; 1 - Response to touch (stroke);  2 - Flees prior to touch |
| **Diarrhea** | 0 – None; 1 - Present |
| **Skin Irritation** | 0 – None; 1 - Present |
| **Positional Passivity** | 0 - Struggles when held by the tail, 1 - Struggles when held by the neck; 2 - Struggles when laid supine; 3 - No struggle |
| **Limb Grasp** | 0 – None; 1 - All 4 limbs clasp in 30 s (note any other form of limb clasping) |
| **Body Tone** | 0 - Flaccid, cavity does not return to normal; 1 - Skin quickly returns to normal |
| **Wire Manoeuvre** | 0- Active grip with hind limbs; 1 - Difficulty to grip with hind limbs; 2 - Unable to grip with hind limbs;  3 – Falls within a few seconds; 4 - Falls immediately |
| **Provoked Biting** | 0 - None, 1- Bites the dowel |
| **Irritability** | 0 - No struggle when restrained; 1 - Struggles upon supine restraint |
| **Aggression** | 0 - No biting/attacks; 1 - Bites the dowel or attacks at any point |
| **Vocalisation** | 0 - No vocalisation; 1 - Vocalisation |
