# Supplementary material for: HDAC4 Reduction: A Novel Therapeutic Strategy to Target Cytoplasmic Huntingtin and Ameliorate Neurodegeneration
Source: PLoS Biol. 2013 Nov 26;11(11):e1001717. doi: 10.1371/journal.pbio.1001717 (PMC3841096; doi:10.1371/journal.pbio.1001717)
Supplement: Table S3 — Summary of the Taq-man assays used in this study. (DOCX) [file pbio.1001717.s006.docx]

| **Assay** | **Gene** | **Description / Source** |
| --- | --- | --- |
| *Hdacs 1-11* | Histone deacetylases 1-11 | Mielcarek et al. 2011 |
| *Bdnf* | Brain-derived neutrophic factor | Benn et al. 2008 |
| *HTT* exon-1 | Exon 1 of human huntingtin | Benn et al. 2008 |
| *HTT* | Mouse huntingtin | Benn et al. 2008 |
| *Yhwaz* | Phospholipase A2 | Primer design |
| *Ubc* | Ubiquitin C | Primer design |
| *Rpl13a* | Ribosomal protein L13a | Primer design |
| *Atp5b* | ATP synthase 5B subunit | Primer design |
| *Nedd4* | Neural precursor cell expressed 4 | Primer design |
| *Eif4g* | Eif4g1 eukaryotic translation initiation factor 4 | Primer design |
| *Cyp39* | Cyp39a1 cytochrome P450 | Primer design |
| *Cd2a* | CD2-associated protein | Primer design |
| *Zch15* | Zinc finger CCCH-type containing 15 | Primer design |
| *Trove6* | TROVE domain family, member 6 | Primer design |
| *Secis* | SECIS binding protein 2-like | Primer design |
| *Peli1* | Pellino 1 | Primer design |
| *Malat1* | Metastasis associated lung adenocarcinoma transcript 1 | Primer design |
| *Lrpb1* | Low density lipoprotein-related protein 1B | Primer design |
| *Enpp5b* | Ectonucleotide pyrophosphatase/phosphodiesterase 5 | Primer design |
| *Casc4* | Cancer susceptibility candidate 4 | Primer design |
