# Supplementary material for: HDAC4 Reduction: A Novel Therapeutic Strategy to Target Cytoplasmic Huntingtin and Ameliorate Neurodegeneration
Source: PLoS Biol. 2013 Nov 26;11(11):e1001717. doi: 10.1371/journal.pbio.1001717 (PMC3841096; doi:10.1371/journal.pbio.1001717)
Supplement: Table S4 — Summary of the antibodies used in this study. WB, western blotting; IP, immunoprecipitation; IHC, immunohistochemistry; SEPRION, seprion ligand ELISA for aggregated huntingtin; TR-FRET, time resolved Förster resonance energy transfer for soluble huntingtin. (DOCX) [file pbio.1001717.s007.docx]

| **Antibody** | **Catalogue**  **number** | **Source** | **Dilution/**  **amount** | **Application** | **Reference** |
| --- | --- | --- | --- | --- | --- |
| HDAC4 | DM-15 | Sigma | 1 in 500 | WB | N/A |
| HDAC4 | DM-15 | Sigma | 1μg | IP | N/A |
| HDAC4 | SC H-92 | Santa Cruz | 1 in 1000 | WB | N/A |
| HDAC4 | H-92 | Santa Cruz | 1μg | IP | N/A |
| HDAC4 | 2072 | Cell Signalling | 1 in 100 | IHC | N/A |
| HDAC5 | Ab56929 | Abcam | 1 in 1000 | WB | N/A |
| HDAC5 | Ab56929 | Abcam | 1 ug | IP | N/A |
| S830 | N/A | In house | 1 in 1000 | WB | Sathasivam et al. 2001 |
| S830 | N/A | In house | 1 in 2000 | Seprion |  |
| S830 | N/A | In house | 1 in 100 | IHC |  |
| MW8 | N/A | P.Patterson | 1 in 2000 | WB | Ko et al. 2001 |
| MW8 | N/A | P.Patterson | 1 in 4000 | Seprion |  |
| 3BH10 | P1874 | Sigma | 1μg | IP | N/A |
| MW1 | N/A | P.Patterson | 1μg | IP | Ko et al. 2001 |
| MW1 | N/A | P.Patterson | 1 ng in 6 μl | TR-FRET |  |
| 2B7 | N/A | Novartis | 10 ng in 6 μl | TR-FRET | Weiss et al. 2009 |
| 2166 | MAB2166 | Sigma | 1μg | IP | N/A |
| α-tubulin | T9026 | Sigma | 1 in 40000 | WB | N/A |
| Histone H3 | Ab1791 | Abcam | 1 in 30000 | WB | N/A |
| α-Goat HRP | P044901 | Dako | 1 in 3000 | WB | N/A |
| α-Mouse HRP | P0260 | Dako | 1 in 3000 | WB | N/A |
| α-Rabbit HRP | 32460 | Pierce | 1 in 20000 | WB | N/A |
| α-Sheep Alexa 555 | A21436 | Invitrogen | 1 in 1000 | IHC | N/A |
| α-Rabbit Alexa 488 | A21438 | Invitrogen | 1 in 1000 | IHC | N/A |
