# Supplementary material for: HDAC4 Reduction: A Novel Therapeutic Strategy to Target Cytoplasmic Huntingtin and Ameliorate Neurodegeneration
Source: PLoS Biol. 2013 Nov 26;11(11):e1001717. doi: 10.1371/journal.pbio.1001717 (PMC3841096; doi:10.1371/journal.pbio.1001717)
Supplement: Text S1 — Supporting materials and methods. Plasmids and GST pull-downs and electrophysiological recordings. (DOCX) [file pbio.1001717.s008.docx]

**SUPPORTING INFORMATION**

**SUPPLEMENTAL MATERIALS AND METHODS**

**Primer and probe sequences for Taqman real-time PCR**

Primer and probe sequences were previously described as follows: *Hdacs* 1-11 [[1](#_ENREF_1)], *Bdnf* and ***exon 1 Htt*** transcripts [[2](#_ENREF_2)] and were purchased from Operon. **Primer and probe sequences for other Taqman assays are listed in Table S3 and were purchased from Primer Design.**

**Plasmids and GST-pull downs**

The pDEST47-HDAC4 expression vector was transcribed and translated *in vitro* in the presence of [^35^S]-methionine in reticulocyte lysate (Promega) according to the manufacturer’s protocol. For expression and purification of GST fusion proteins, overnight cultures of *E. coli* expressing the recombinant GST plasmids were diluted 1:10 and after 1 h of culture, isopropyl β-D-thiogalactoside (0.1 mM final concentration) was added. After 3 h, bacteria were collected by centrifugation at 3,500 x *g*, resuspended 1:10 in NETN (0.5% Nonidet P-40; 1 mM EDTA; 20 mM Tris pH 8.0; 125 mM NaCl) containing complete protease inhibitors (Roche) with 10 mM dithiothreitol and 1 mM phenylmethylsulfonyl fluoride, sonicated, and centrifuged. Fusion proteins were purified from the supernatant on glutathione-Sepharose beads (GE Healthcare) by incubating 250 µl of GST-beads (1:1 in NETN) per 1 ml bacterial supernatant for 30 min. Finally the beads were washed 3x with 10 ml NETN buffer prior to the interaction assay.

For the GST-pull down 1 µl of [^35^S]-labelled HDAC4 was incubated at 4°C with either 50 µl (1:1 in NETN) GST, GST-Exon1 20Q, or GST-Exon1 53Q beads overnight in a final volume of 1 ml NETN on a rotating wheel. Samples were centrifuged at 13,000x *g* for 10 sec and the beads washed 4x with 1 ml cold NETN buffer. After the final wash, the beads were dried in a speed-vac for 30 min, re-suspended in 10 µl 2x Laemmli buffer. GST complexes were eluted by denaturation at 75°C for 5 min, and separated by 10% SDS–PAGE. Finally, the gels were fixed (30% Methanol, 10% Acetic acid), stained with coomassie brilliant blue and amplified (Amplify, GE Healthcare) prior to gel drying. The ^[35]^S-labelled proteins were visualised by fluorography.

**Electrophysiological Recordings**

**Acute striatal slice preparation**

250 µm thick horizontal corticostriatal brain slices were acutely prepared on a VT1200S vibrotome (Leica) [[3](#_ENREF_3)] from 7-8 week (6/group) and 12 week old (2/group) WT, R6/2, *Hdac4*HET and Dble mice in ice-cold sucrose solution composed of (in mM): sucrose 248, KCl 2, MgSO_4_ 2, NaH_2_PO_4_ 1.25, NaHCO_3_ 26, D-glucose 11.1, kynurenic acid 1. Slices containing the dorsal striatum were transferred into an incubation chamber containing artificial cerebrospinal fluid (ACSF) with a composition of (in mM): NaCl 125, KCl 2.5, CaCl_2_ 2, MgCl_2_ 1, NaHCO_3_ 25, NaH_2_PO_4_ 1.25, D-glucose 25 at room temperature (RT) for at least 30 min to recover. All solutions were continuously bubbled with carbogen gas (95% CO_2_, 5% O_2_). For recordings, the slices were transferred into a 1 ml volume chamber and continuously superfused with ACSF (~1.5 ml/min) at RT.

**Patch-clamp recordings**

Medium spiny neurons within the slice were visualized using an upright microscope equipped with infrared differential interference contrast (IR-DIC) and a 60x water-immersion objective (all from Olympus). Cells with a somatic size smaller than about 15 µm were selected (average capacitance (C_m_)=63±1 pF, n=122), and recordings with an unstable access resistance (R_a_) (>10% change over the period of recording, or initial R_a_ larger than 20 MΩ, were discarded (average R_a_=9 ± 0.26 MΩ), no significant difference between groups). Between 15-20 neurons for each group were recorded in the 7–8 week datasets, and 11-13 neurons per group in the 12 week datasets.

Whole-cell patch clamp experiments were performed in both voltage-clamp and current clamp mode using a software-controlled MultiClamp 700B amplifier in combination with a Digidata 1440A digitizer (Molecular Devices). Patch pipettes were pulled from borosilicate glass capillaries (electrode resistance=2-4 MΩ). For recordings of MSN membrane parameters and evoked corticostriatal transmission, the intracellular solution contained (in mM): K-gluconate 105, KCl 30, MgCl_2_ 4, EGTA 0.3, HEPES 10, Na_2_ATP 4, Na_3_GTP 0.3 and Na-phosphocreatine 10, pH 7.35. Under voltage clamp control, cells were held at -80 mV and C_m_ and membrane resistance (R_m_) were calculated in response to a -10 mV hyperpolarizing pulse using the membrane test function of pClamp. Resting membrane potential (RMP), rheobase (minimum current injection required to elicit an action potential) and resultant action potential amplitude of the neurons were then evaluated by switching to current clamp mode, prior to returning to voltage clamp control. Cortical stimulation to evoke glutamatergic excitatory post synaptic currents (eEPSCs) in the striatal MSNs were performed via a custom-built bipolar tungsten electrode that was placed between layer V of the cortex and deeper cortical layers. In a given MSN recording, cortical stimulation at a range of intensities (0.2–20 V) was used to determine the strength of glutamatergic transmission. Stimulation was repeated 20 times at a frequency of 0.1 Hz for each intensity level and the resultant eEPSCs averaged per neuron. To determine the paired-pulse ratio, two stimulating pulses with a 20 ms inter stimulus interval (ISI) were applied to the cortex (at 2-3 times the intensity needed to evoke a minimal response). Each individual cell was stimulated 5 times at a frequency of 0.1 Hz. The paired-pulse ratio was then calculated based on the average of these responses by dividing the amplitude of the second postsynaptic current recorded from the MSN with the first.

A separate set of experiments were performed in 7-8 week old mice (n=2/group) to isolate miniature excitatory postsynaptic currents (mEPSCs). In this instance, the intracellular solution comprised (in mM): Cs-methanesulfonate 100, Na-methanesulfonate 10, QX314-Cl 5, TEA-Cl 10, HEPES 10, CaCl_2_ 1, EGTA 10, MgATP 5, Na_3_GTP 0.5, pH 7.2, and all recordings were performed with the addition of tetrodotoxin (TTX, 0.5 µM) and picrotoxin (50 µM) to the ACSF. Recordings were made at -80 mV for 20 min after observing stable activity for 10 min. The experiments were terminated by application of 10 µM CNQX to confirm that the events recorded were entirely AMPA receptor mediated. Between 11-12 neurons/group were recorded.

**Analysis of electrophysiological data**

For all recordings, data were low-pass filtered at 2 kHz before being sampled at 10 kHz using Clampex 10 (Molecular Devices). Data were analyzed with ClampFit 10 (Molecular Devices), Prism 5 (GraphPad) and Igor Pro 6 (Wavemetrics).

mEPSCs recordings were analyzed for amplitude and frequency using Minianalysis (Synaptosoft). After automated mEPSC detection, each trace was checked for false positives manually. Statistical analysis was performed using Prism 5 and Igor Pro 6 One-way ANOVA with *post hoc* Tukey’s test was used to assess statistical significance. To perform a Kolmogorov Smirnov (KS) test on the mEPSC recordings, cumulative plots were first created by pooling the inter-event interval and amplitude data for each set (n=12 for WT, HDAC and DBL and n=11 for R6/2).

**REFERENCES**

1. Mielcarek M, Benn CL, Franklin SA, Smith DL, Woodman B, et al. (2011) SAHA Decreases HDAC 2 and 4 Levels In Vivo and Improves Molecular Phenotypes in the R6/2 Mouse Model of Huntington's Disease. Plos One 6.

2. Benn CL, Fox H, Bates GP (2008) Optimisation of region-specific reference gene selection and relative gene expression analysis methods for pre-clinical trials of Huntington's disease. Mol Neurodegener 3: 17.

3. Fino E, Glowinski J, Venance L (2005) Bidirectional activity-dependent plasticity at corticostriatal synapses. Journal of Neuroscience 25: 11279-11287.
